# The Effect of 1,2,4-Triazole-3-thiol Derivatives Bearing Hydrazone Moiety on Cancer Cell Migration and Growth of Melanoma, Breast, and Pancreatic Cancer Spheroids

**DOI:** 10.3390/ph15081026

**Published:** 2022-08-20

**Authors:** Aida Šermukšnytė, Kristina Kantminienė, Ilona Jonuškienė, Ingrida Tumosienė, Vilma Petrikaitė

**Affiliations:** 1 Department of Organic Chemistry, Kaunas University of Technology, Radvilėnų pl. 19, 50254 Kaunas, Lithuania; 2 Department of Physical and Inorganic Chemistry, Kaunas University of Technology, Radvilėnų pl. 19, 50254 Kaunas, Lithuania; 3 Laboratory of Drug Targets Histopathology, Institute of Cardiology, Lithuanian University of Health Sciences, Sukilėlių pr. 13, 50162 Kaunas, Lithuania

**Keywords:** hydrazone, isatin, 2-oxindole, 1,2,4-triazole, melanoma, pancreatic tumor, triple-negative breast cancer, 3D tumor model, migration

## Abstract

4-Phenyl-3-[2-(phenylamino)ethyl]-1*H*-1,2,4-triazole-5(4*H*)-thione was used as a starting compound for the synthesis of the corresponding 1,2,4-triazol-3-ylthioacetohydrazide, which reacts with isatins and various aldehydes bearing aromatic and heterocyclic moieties provided target hydrazones. Their cytotoxicity was tested by the MTT assay against human melanoma IGR39, human triple-negative breast cancer (MDA-MB-231), and pancreatic carcinoma (Panc-1) cell lines. The selectivity of compounds towards cancer cells was also studied. In general, the synthesized compounds were more cytotoxic against the melanoma cell line. *N*′-(2-oxoindolin-3-ylidene)-2-((4-phenyl-5-(2-(phenylamino)ethyl)-4*H*-1,2,4-triazol-3-yl)thio)acetohydrazide, *N*′-((1*H*-pyrrol-2-yl)methylene)-2-((4-phenyl-5-(2-(phenylamino)ethyl)-4*H*-1,2,4-triazol-3-yl)thio)acetohydrazide and *N*′-(2-hydroxy-5-nitrobenzylidene)-2-((4-phenyl-5-(2-(phenylamino)ethyl)-4*H*-1,2,4-triazol-3-yl)thio)acetohydrazide were identified as the most active among all synthesized compounds in 3D cell cultures. *N*′-(4-(dimethylamino)benzylidene)-2-((4-phenyl-5-(2-(phenylamino)ethyl)-4*H*-1,2,4-triazol-3-yl)thio)acetohydrazide inhibited all cancer cell migration, was characterized as relatively more selective towards cancer cells, and could be further tested as an antimetastatic candidate.

## 1. Introduction

Cancer is a major global public health problem and is growing as the leading cause of mortality, accounting for vast morbidity worldwide. It is a malignant disease characterized by rapid and uncontrolled cell proliferation [1]. Pancreatic carcinoma is associated with one of the worst clinical outcomes due to its aggressive, metastatic, and drug-resistant nature. Pancreatic cancer accounts for 7% of all cancer deaths [2]. Breast cancer is the leading cause of cancer morbidity, disability, and mortality in women worldwide. It was the most diagnosed malignancy in 2020 [3]. Among skin cancers, melanoma is the most lethal form, accounting for 75% of deaths due to skin cancer, although it is diagnosed only in 4% of skin cancer cases [4]. 

Cytotoxic drugs are among the most important treatments used for cancer; however, their permeability and efficiency are low. Furthermore, a variety of anticancer cytotoxic drugs show different adverse side effects due to the low selectivity of the antiproliferative action and allow tumors to develop resistance to multiple chemotherapeutic drugs [5]. The search for new effective anticancer agents with superior selectivity towards cancer cells is of crucial importance [6]. Small-molecule kinase inhibitors have been widely investigated as novel anticancer therapeutics. Kinases are enzymes that transfer a phosphate group to a protein and regulate various cellular functions such as proliferation, differentiation, migration, metabolism, and angiogenesis by activating several signalling pathways [7]. Kinases have also been frequently found to be deregulated and overexpressed in cancerous tissues. Therefore, modification of the kinase activity by employing small molecules has emerged as a strategic approach for cancer treatment [8].

1,2,4-Triazole is one of the most significant nitrogen-containing scaffolds in the field of medicinal chemistry due to its diverse biological properties, including anticancer activity [9,10,11,12]. Among the azoles, triazoles are the most stable compounds and are difficult to cleave. 1,2,4-Triazoles act as important pharmacophores by interacting with biological receptors with high affinity due to their dipole character, hydrogen bonding capacity, rigidity, and solubility [13]. The 1,2,4-triazole nucleus is stable to metabolism and acts as an important pharmacophore by interacting at the active site of a receptor as a hydrogen bond acceptor and as a donor. Due to its polar nature, the triazole nucleus can increase the solubility of the ligand and significantly improve the pharmacological profile of the drug [14]. Many anticancer agents such as fluconazole, tebuconazole, triadimefon, and ribavirin bear a 1,2,4-triazole moiety, revealing their potential in the development of novel anticancer agents [15]. Triazole heterocycles that incorporate sulfur in the form of mercapto- and thione-substitution show more potency compared to their parent derivatives [16]. A variety of biological activities, such as anticancer, antimicrobial, antitubercular, hypoglycemic, etc., properties have been reported for a large number of 1,2,4-triazole-3-thione and 1,2,4-triazole-3-thiol derivatives [17,18,19].

Hydrazone derivatives bearing various heterocyclic scaffolds have gained significant importance in medicinal chemistry because of their diverse biological properties including anticancer, antioxidant, antimicrobial, anticonvulsant, anti-inflammatory, etc., activities [20,21,22,23,24]. Hybrid derivatives of isatin bearing the hydrazone moiety have been shown to exhibit good activity against various tumour cells (colon, leukemia, breast, and kidney) [25].

Isatin derivatives have been widely recognized in cancer therapeutics as protein kinase inhibitors. Sunitinib is an oxindole-containing clinically used drug for the treatment of renal cell carcinoma [26]. Another derivative of oxindole, nintedanib, was approved in 2020 in the United States for the treatment of interstitial lung diseases such as idiopathic pulmonary fibrosis and chronic fibrosis with a progressive phenotype. It has an effective antiproliferative feature, inhibiting angiokinase and restricting growth factor, consequently being amongst the most potent indolinone compounds [8]. Many researchers have exploited the isatin moiety taking advantage of NH in the first position, the carbonyl position C2, and C3 for the synthesis of various derivatives that possess different biological activities including anticancer effects in different types of cancer [27,28,29]. Diverse substitution at the C5 position has been associated with the promising activity of synthesized oxindole derivatives such as progesterone antagonist, vasopressin antagonist, anti-Alzheimer, phosphate inhibitor, kinase inhibitor, neuroprotection, NMDA blocking, and anticancer, antioxidant, antibacterial, and anti-HIV activity [30].

Hybrid isatin-triazole hydrazones have been reported as potent microtubule affinity-regulating kinase 4 inhibitors based on in vitro anticancer tests performed on MCF-7, MDA-MB-435s, and HepG2 cells [31].

Based on the pharmacological implications of the moieties discussed above and as a continuation of our search for biologically active hybrid compounds that carry heterocyclic fragments [32,33,34,35], we report herein the synthesis of a series of 1,2,4-triazole-3-thiol derivatives bearing 5-substituted 2-oxindole-hydrazone moieties. Another series of hybrid compounds includes hydrazones bearing a 1,2,4-triazole-3-thiolyl moiety along with various aromatic and heterocyclic fragments. Hybrid compounds that contain pyrimidine, pyrrole, and pyrazole moieties have been extensively shown to possess antiproliferative properties [36,37,38,39,40,41].

Three human cancer cell lines, namely melanoma (IGR39), triple-negative breast cancer (MDA-MB-231), and pancreatic carcinoma (Panc-1) were chosen for this study. These tumour types are characterized as very aggressive and invasive and are usually resistant to available chemotherapeutics [42,43,44]. In order to reduce the development of cancer resistance, existing anti-cancer drugs are often combined with novel therapeutics that could improve their penetration into cancer cells, reduce the efflux from the cells, or improve the effectiveness of cancer therapy by affecting different metabolic pathways [45]. Kinase inhibitors are one of the drug groups that are used in combinatorial studies [46]. We decided to explore novel compounds in 3D cell cultures (tumor spheroids) and migration assays, and to identify the most promising ones for further development.

## 2. Results and Discussion

### 2.1. Chemistry 

**Scheme 1:**
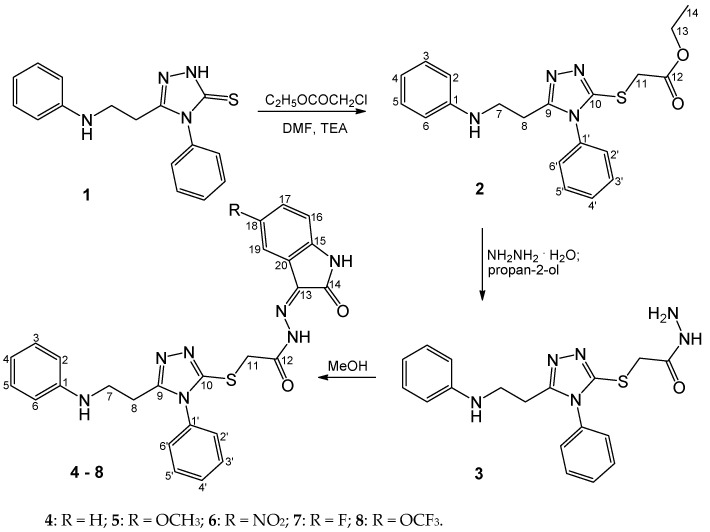
Synthesis of hydrazones **4**–**8**.

**Scheme 2:**
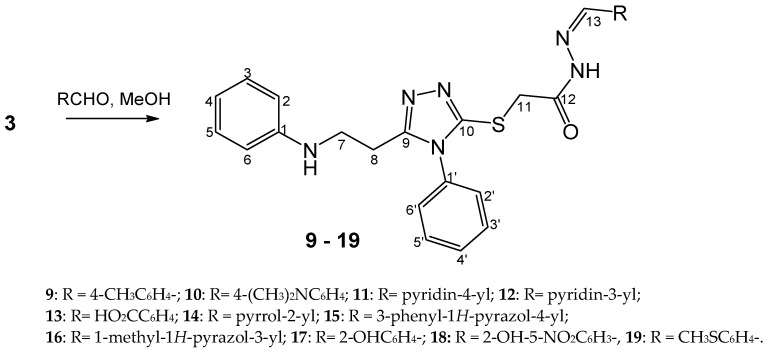
Synthesis of hydrazones **9**–**19**.

The target hydrazones **4–19** were synthesized according to the previously reported synthetic route [33,35] as shown in Scheme 1 and Scheme 2. Reaction of 1,2,4-triazole-5-thione **1** [47] with ethyl chloroacetate in DMF in the presence of triethylamine at room temperature provided ethyl 2-[[4-phenyl-5-[(phenylamino)ethyl]-4*H*-1,2,4-triazol-3-yl]thio]acetate (**2**) in 80% yield. The subsequent reaction of **2** with hydrazine hydrate in propan-2-ol at 60 °C gave 1,2,4-triazol-3-ylthioacetohydrazide **3** in 94% yield (Scheme 1). 

The formation of ethyl ester **2** is confirmed by the typical ^1^H NMR resonances ascribed to the protons of methyl group (H_14_) at 1.19 ppm and methylene group (H_13_) at 4.11 ppm as well as a singlet at 4.03 ppm attributed to the methylene group protons (H_11_). In the ^13^C NMR spectrum for **2**, the resonance at 154.13 ppm attributed to the C_10_ carbon is shifted upfield in respect of the thione group carbon (C_10_, 166.60 ppm) in the ^13^C NMR spectrum of the precursor molecule **1** [47]. In the ^1^H NMR spectrum of **3**, the singlets at 4.24 ppm and 4.29 ppm (integrated for two protons) and 9.32 ppm (integrated for one proton) are attributed to the protons of the NH_2_ and NH groups, respectively, in the hydrazide moiety. 

2-Oxindole derivatives **4**–**8** were synthesized from hydrazide **3** and the corresponding isatins in methanol under reflux in good yields (63–75%) [35]. In the ^1^H NMR spectra of **4**–**8**, the proton of the secondary amine group adjacent to the C_1_ carbon resonated as a singlet at 5.65 ppm (Figures S10, S13, S16, S19, and S22 in the Supplementary Materials). The proton in the amide group (NHC_12_) resonated in the range of 11.5–13.4 ppm, while the proton singlets at the higher field in the range of 10.8–11.9 ppm are attributed to the secondary amine group in the 2-oxindole moiety. The ^1^H NMR spectra of hydrazones **4**–**8** display double sets of resonances of the NHC_12_ protons, and a 2-oxindole NH proton due to restricted rotation around the amide bond (Figures S10, S13, S16, S19, and S22 in the Supplementary Materials). This splitting of the proton resonances indicates that in DMSO-*d_6_*, hydrazones exist as a mixture of *Z/E* isomers with respect to the hindered rotation around the amide bond. Usually, the *Z* isomer predominates [32,33]. In the ^1^H NMR spectra, the NH protons of *Z* isomers resonate in a lower field with respect to the resonances attributed to *E* isomers [48]. Two carbonyl group carbon resonances (C_14_ and C_12_, respectively) in the range of 162.6–174 ppm confirm the presence of the 2-oxindole moiety along with the amide group in molecules **4**–**8** (Figures S11, S14, S17, S20, and S23 in the Supplementary Materials).

Hydrazone derivatives **9**–**19** were synthesized from hydrazide **3** and the corresponding aldehydes in methanol under reflux in the yield range of 29–98% (Scheme 2) [35,49]. The structures of the synthesized compounds have been confirmed by ^1^H and ^13^C NMR, IR, and MS data. In the ^1^H NMR spectrum for **15** (Figure S43 in the Supplementary Materials), multiplets at 7.88–8.25 integrated for two protons are attributed to the H_13_ proton and the one in the CH group of the pyrazole ring. In the ^1^H NMR spectrum for **16** (Figure S46 in the Supplementary Materials), two protons in the pyrazole ring resonated as multiplets at 6.52–6.65 ppm and 7.35–8.18 ppm (this signal overlaps with the resonances of aromatic protons and the one of the H_13_ proton). The presence of the methyl group attached to the N atom in pyrazole moiety was confirmed by the singlet at 3.40 ppm. The resonance attributed to the C_11_ carbon atom in the ^13^C NMR spectra of hydrazones **9**–**19** was shifted downfield by ~1 ppm compared to the respective resonance in the ^13^C NMR spectrum of **2** (Figures S26, S29, S32, S35, S38, S41, S44, S47, S50, S53, and S56 in the Supplementary Materials). The ^13^C NMR spectra of **13**–**19** display double sets of C_11_ carbon resonances owing to the formation of *Z/E* isomers in in the DMSO-*d_6_* solutions (Figures S38, S41, S44, S47, S50, S53, and S56 in the Supplementary Materials). 

### 2.2. Pharmacology

#### 2.2.1. Cytotoxicity

The synthesized compounds showed different activity against human cancer cell lines at 50 µM concentration. This concentration was chosen based on the solubility of the compounds and our previous experience. It has been shown that the higher concentrations of 50–100 µM allow distinguishing the most active compounds [33,50] and is often used for a primary screening of anticancer agents [51]. Our calculated EC_50_ values correspond to the 50% lethal concentration (LC_50_), which is calculated by the US National Cancer Institute (NCI) during screening of compounds [52]. However, NCI performs screening on cancer cell panels, and does not evaluate the compound effects on normal cells (e.g., fibroblasts); thus, the obtained results usually lack information about compound selectivity towards tumours. In addition to compound screening in the cell monolayer, NCI recently has introduced the hollow fibre assay, which includes a short-term in vitro culture (up to 48 h) of a panel of 12 human tumour cell lines in fibres, and the results obtained well represent compound activity in tumour xenografts [53]. Thus, we also decided to first select the most promising candidates by the MTT assay and then test their activity in tumour spheroids.

**Figure 1:**
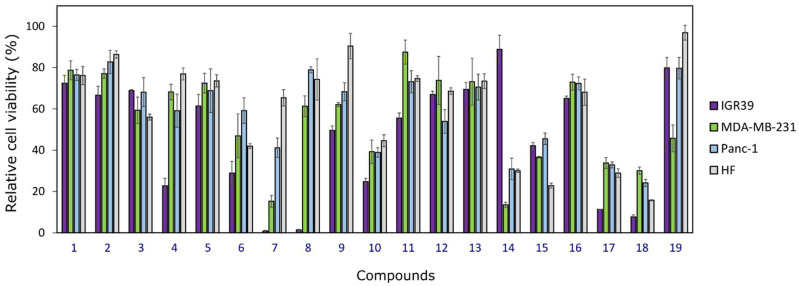
Effect of hydrazones **1–19** on cancer cell viability at a concentration of 50 µM against human melanoma IGR39, human triple-negative breast cancer MDA-MB-231, human pancreatic carcinoma Panc-1 cell lines, and human fibroblasts HF; *n* = 3.

Surprisingly, the synthesized hydrazones showed relatively high activity against the IGR39 cell line used in the screening experiments (Figure 1), except compounds **1**, **2**, **3**, **5**, **12**, **13**, **14**, **16**, and **19**. This type of cancer (malignant melanoma) is usually considered as a relatively resistant one due to many different resistance mechanisms [54]. Triple-negative breast cancer cells MDA-MB-231 and pancreatic cancer cells Panc-1 were less sensitive to a majority of the tested compounds. In general, triple-negative breast cancer cells are characterized by their high resistance to many chemotherapeutic drugs due to high expression of P-glycoprotein, the stemness properties, and other mechanisms [55]. Pancreatic cancer is one of the most difficult cancer types to treat and, therefore, is characterized by high resistance to many available anticancer agents [56]. 

**Figure 2:**
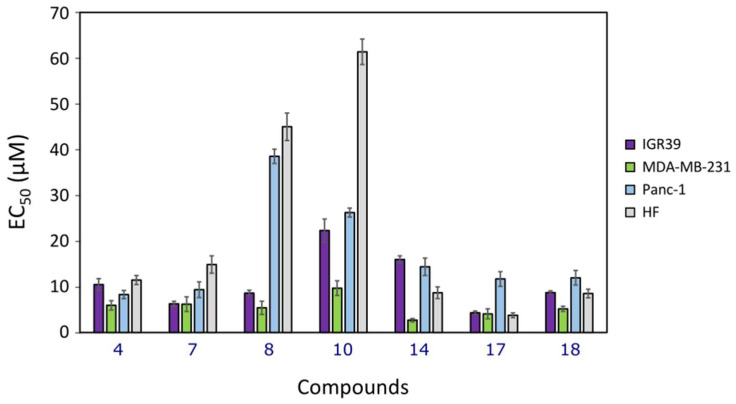
EC_50_ values of the most active hydrazones **4, 7, 8, 10, 14, 17** and **18**, obtained by the MTT assay, *n* = 3.

In general, among the synthesized 1,2,4-triazole-3-thiol derivatives, hydrazones 1**7** and **18** bearing 2-hydroxybenzene or 2-hydroxy-5-nitrobenzene moiety, respectively, were identified as the most active ones against all tested cancer cell lines. Meanwhile, hydrazones bearing 5-fluoro- or 5-trifluoromethoxy-2-oxindole moiety **7** and **8**, respectively, were the most active among the synthesized compounds against the IGR39 cell line. Interestingly, the least active derivatives against IGR39 (compounds **14** and **19**) showed relatively higher activity towards the triple-negative breast cancer cell line. These compounds contain pyrrole and 4-(methylthio)benzene fragments, respectively, in their structure, which could be a key element to obtain a selectivity against the MDA-MB-231 cell line. Two more compounds, **4** and **10**, showed a moderate selectivity against the melanoma IGR39 cell line; therefore, they were also included in further studies. In total, seven of the most active compounds (**4**, **7**, **8**, **10**, **14**, **17**, and **18**) were selected for further studies, and their effective concentrations that reduce cell viability by 50% (EC_50_ values) were determined (Figure 2). 

Hydrazone **10** bearing *p*-(dimethylamino)benzene moiety has been shown to be the least active out of seven selected compounds, but it was most selective towards cancer cell lines (EC_50_ = 22.3 ± 2.5 µM against IGR39, EC_50_ = 9.7 ± 1.6 µM against MDA-MB-231, and EC_50_ = 26.2 ± 1.0 µM against the Panc-1 cell line) compared to fibroblasts (EC_50_ = 61.4 ± 2.8 µM). The cytotoxic effects of hydrazone bearing the 5-trifluoromethoxy-2-oxindole moiety **8** against Panc-1 were the lowest among the seven tested compounds (EC_50_ = 38.5 ± 1.6 µM). However, its activity against melanoma and breast cancer cells was comparable to that of the other most active compounds. Furthermore, compound **8** showed a lower cytotoxicity against fibroblasts, which makes it more promising. 

Tiago et al. [57] have shown that the presence of stroma may enhance the drug resistance of melanoma in vitro, due to the interaction between tumour and stroma, which means that selectivity in cell monolayers does not represent the in vivo situation quite well. Cancer-associated fibroblasts are known to also change the resistance of cancer cells in monolayers to chemotherapy [58]. Furthermore, many studies have indicated not very large differences between cytotoxicity against normal and cancer cells, and there are still some ongoing debates on how to interpret the obtained results [59] and what selectivity index could be considered as “good“ enough. Usually, the higher toxicity towards cancer cells at a value twice or lower than towards normal cells, is considered to be already showing selectivity [60]. In our scenario, compounds **8** and **10** were even more selective against several cancer cell lines. 

All seven compounds showed higher cytotoxicity towards malignant melanoma IGR39 cell lines, especially pyrrole derivative **17**, and they could be further developed as anti-melanoma drugs. Dacarbazine, used to treat melanoma, is much less active than these compounds and inhibits melanoma A375 cell proliferation at concentrations of 25–100 μM [61]. There are limited studies on compounds against the IGR39 cell line, which may show the importance of our study. In our previous experiments on new sunitinib derivatives, we identified several highly active derivatives 4001, 4007, and 4008 that possessed an antiproliferative effect at 140–500 nM concentrations [34]. However, the BRAF inhibitor dabrafenib, which is used as a chemotherapeutic drug for melanoma treatment, reduces melanoma cell viability at a nanomolar concentration [62].

#### 2.2.2. Effect on Cell Migration

The ‘Wound healing’ assay was used to evaluate compound activity on cell migration. The most active compounds **4**, **7**, **8**, **10**, **14**, **17**, and **18** were tested for their effect on human melanoma IGR39, human triple-negative breast cancer MDA-MB-231, and pancreatic carcinoma Panc-1 cell migration at 10 µM concentration. The effect of this concentration on cell viability after 24 h, 48 h and 72 h was studied in separate experiments (Figures S58–60) using the MTT assay (the procedure is described in Supplementary Materials).

**Figure 3:**
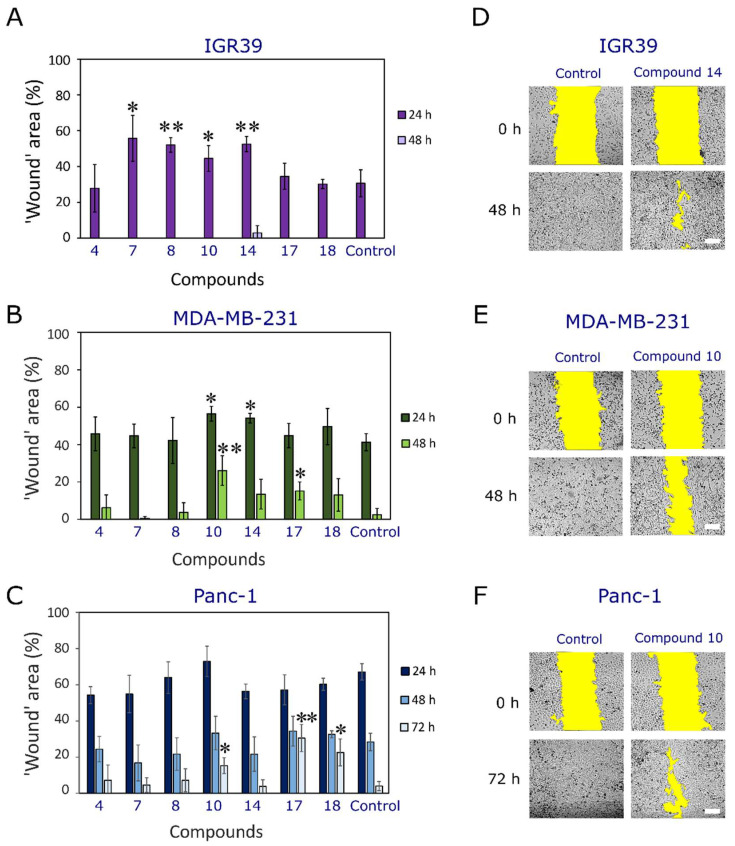
Effect of hydrazones **4**, **7**, **8**, **10**, **14**, **17**, and **18** on human malignant melanoma IGR39 (**A**), human triple-negative breast cancer MDA-MB-231 (**B**), and human pancreatic carcinoma (Panc-1) (**C**) cell migration; *n* = 3. Photos of the ‘wound’ area (marked in yellow) in IGR39 (**D**), MDA-MB-231 (**E**), and Panc-1 (**F**) monolayers at the beginning and the end of the experiment. Scale bar indicates 100 µm. The asterisks (*) indicate *p* < 0.05 and (**) indicate *p* < 0.01 compared to the control (untreated cells).

Several compounds have been shown to possess an effect on tested cancer cell migration (Figure 3). In general, the effect on IGR39 cell migration was more strongly expressed after a shorter duration (24 h) of incubation, while the effect on the pancreatic cancer Panc-1 cell migration was revealed only after 72 h of incubation. The MDA-MB-231 cells migrated slower both after 24 h and 48 h of incubation with the tested compounds **10**, **14**, and **17**. Hydrazone bearing 2-oxindole moiety **4** did not possess activity either after 24 h or 48 h of incubation with all tested cell lines, compared to the control (*p* > 0.05). Compounds **10** and **14** were identified as the most active compounds; they reduced cell migration from 1.5 to 3 times (Figure 3). 

The compounds did not reduce viability of cells by more than up to 80% after 48 h of incubation, except the compounds **14** and **17** (IGR39 cell viability after 48 h of incubation with these compounds was (67.9 ± 1.8)% and (76.0 ± 7.2)% (Figures S58–60 in Supplementary Materials). The viability of Panc-1 cells after 72 h was reduced up to (74.3 ± 3.4)% by compound **4**, and up to (79.7 ± 3.8)% by compound **17**. In general, there was no correlation between cell viability and cell migration at 10 µM concentration, so it could not be concluded that the migration inhibition effect was the result of reduced cell proliferation. Of course, compound effects on cell proliferation cannot be excluded, but we assume that there could be many still unknown effects on cell metabolic pathways more specifically related to the migration inhibition, and the reduction in cell viability could be a final consequence of these effects. 

Malignant melanoma and triple-negative breast cancer cell lines are characterized by high aggressiveness and invasiveness. As a result, in order to combat the resistance, an approach of inhibiting cancer cell migration has been widely studied [63,64]. There is a need for more effective novel compounds with a potential to reduce cell migration. One of the possible solutions could be a combination strategy where the new compound or drug is added in combination with already clinically used highly effective anticancer agents [65]. Mishra et al. [66] determined that the pan-PI3K inhibitor combined with the widely used anticancer drug doxorubicin suppresses cancer cell growth, proliferation, and migration. Inhibition of protein kinase B (AKT) and SRC in the presence of gemcitabine significantly reduced the metastatic potential of pancreatic cancer cells by suppressing the phosphorylation of mTOR and ERK in cells [67]. The synthesized compounds contain fragments characteristic of kinase inhibitors, and they are expected to possess a similar mechanism of action. However, more detailed studies are needed to prove their mechanism of action.

#### 2.2.3. Activity in 3D Cell Cultures (Spheroids)

**Figure 4:**
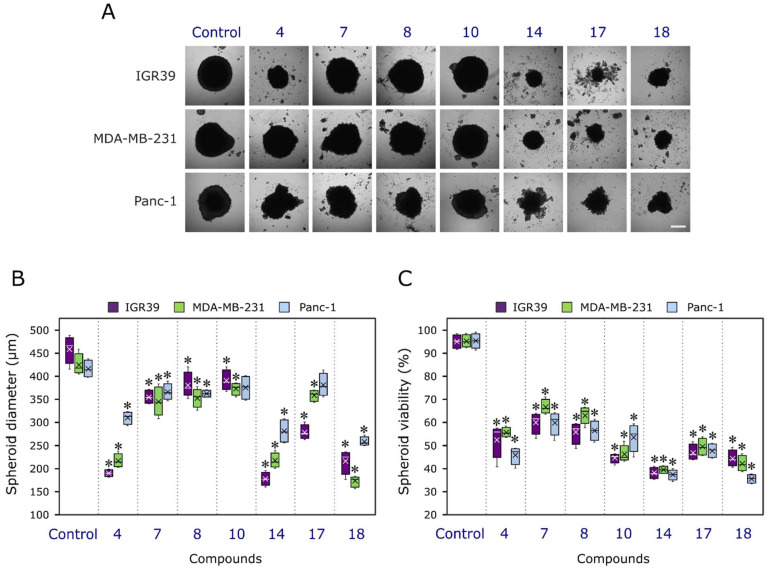
Effect of the most active hydrazones **4**, **7**, **8**, **10**, **14**, **17**, and **18** on 3D cell cultures. (**A**) Photos of human melanoma IGR39, human triple-negative breast cancer MDA-MB-231, and human pancreatic carcinoma Panc-1 tumour spheroids at the end of the experiment (after 8 days of incubation with 10 µM of compounds). (**B**) Spheroid size at the end of the experiment. (**C**) Cell viability in the IGR39, MDA-MB-231, and Panc-1 spheroids. Asterisks (*) indicate *p* < 0.05 compared to the control (untreated spheroids), crosses (×) indicate means; inner dashes indicate medians; whiskers indicate maximum and minimum values. Scale bars indicate 200 µm.

For decades, 3D cell cultures have been widely used as a model to test the anticancer activity of novel substances. This model is considered to represent the real tumour microenvironment much better compared to conventional cell monolayers (2D models). Tumour spheroids are one of the most simplified 3D cell models, and are characterised by hypoxia formation in their core as well as the gradient of tested substances [68]. The effects of 10 µM solutions of the seven selected hydrazones **4**, **7**, **8**, **10**, **14**, **17**, and **18** on melanoma IGR39, triple-negative breast cancer MDA-MB-231 and pancreatic carcinoma Panc-1 cell spheroid growth were evaluated (Figure 4). As shown in Figure 4A, the most active compounds against all three types of spheroids were **14**, **17**, and **18**. Melanoma spheroid growth was mostly inhibited by compounds **4**, **14**, and **18**; IGR39 spheroids began to disintegrate after 8 days of incubation with compound **17**. Meanwhile, Panc-1 spheroids were more resistant to compound treatment, but their growth delay was also observed after incubation with almost all compounds tested (Figure 4B).

It has already been proven by different research groups that measuring only the size of spheroids is not enough to judge about the effectiveness of the compound. The size of the spheroid does not necessarily correlate with the cell viability in 3D [69]; therefore, in our study we also tested cell viability in all spheroids at the end of the experiment (Figure 4C). Interestingly, cell viability was from 1.5-fold to 2.8-fold lower compared to the control in all types of spheroids, despite the size of spheroids being not so different from that of the control group. Such a phenomenon could be explained by the different spheroid morphology due to distinct hypoxic properties and variant gradient of the compounds tested. Several studies have already shown that kinase inhibitors might reduce spheroid size by strengthening cell-cell interaction [70]. On the contrary, Aihara et al. [71] discovered that LATS kinase inhibitors block the Hippo signalling pathway and could promote cell proliferation under 3D culture conditions. The study only shows that kinase inhibitors may possess different effects depending on their specific metabolic pathways, and more extensive studies are needed.

In summary, hydrazones **4**, **14**, and **18** were identified as the most promising anticancer agents out of a series of 1,2,4-triazole-3-thiol derivatives. They were shown to possess a moderate cytotoxicity against the tested cancer cell lines (EC_50_ values were in the range of 2–17 µM). Although these derivatives are not very selective against all cancer cells compared to fibroblasts, yet they were several times more cytotoxic against the triple-negative breast cancer cell line, which is characterized as very invasive and lacking specific targets for chemotherapeutics. Furthermore, selected compounds showed higher or comparable activity to dacarbazine and erlotinib, drugs already approved to treat malignant melanoma and pancreatic cancer. Furthermore, *N*′-(4-(dimethylamino)benzylidene)-2-((4-phenyl-5-(2-(phenylamino)ethyl)-4*H*-1,2,4-triazol-3-yl)thio)acetohydrazide (**10**) was identified as relatively selective towards cancer cells and showed promising results in migration assays on MDA-MB-231 and Panc-1 cells. It is worth exploring its effects on cell migration and invasion in more sophisticated methods such as 3D invadopodia formation or cell migrations in spheroid models and identifying specific pathways that contribute to these effects. The development of antimetastatic agents is facing major challenges [72], and metastasis often leads to death from more aggressive types of cancer, which only proves the need to search for novel effective migrastatic agents.

## 3. Materials and Methods

### 3.1. Chemistry 

#### 3.1.1. Chemical Reagents and Instruments

Reagents were purchased from Sigma-Aldrich (St. Louis, MO, USA) and TCI Europe N.V. (Zwijndrecht, Belgium). The reaction course and purity of the synthesized compounds were monitored by TLC using aluminium plates precoated with silica gel 60 F254 (MerckKGaA, Darmstadt, Germany). The melting points were determined on a MEL-TEMP (Electrothermal, A Bibby Scientific Company, Burlington, NJ, USA) melting point apparatus and are uncorrected. FT-IR spectra (ν, cm^−1^) were recorded on a Perkin–Elmer Spectrum BX FT–IR spectrometer using KBr pellets. The ^1^H and ^13^C-NMR spectra were recorded in DMSO-*d_6_* on a Bruker Avance III (400 MHz, 101 MHz) spectrometer operating in the Fourier transform mode. Chemical shifts (*δ*) are reported in parts per million (ppm) calibrated from TMS (0 ppm) as an internal standard for ^1^H NMR, and DMSO-*d_6_* (39.43 ppm) for ^13^C NMR. Mass spectra were obtained on a Bruker maXis UHR-TOF mass spectrometer (Bruker Daltonics, Bremen, Germany) with ESI ionization.

#### 3.1.2. 4-Phenyl-5-(2-(phenylamino)ethyl)-2,4-dihydro-3*H*-1,2,4-triazole-3-thione (**1**): 

The compound was prepared as described in [47]. M.p., ^1^H and ^13^C NMR spectra as well as IR spectra were found to be identical with those described in [47].

#### 3.1.3. Ethyl 2-((4-phenyl-3-(2-(phenylamino)ethyl)-4,5-dihydro-1*H*-1,2,4-triazol-5-yl)thio)acetate (**2**):

To **1** (4 g, 14 mmol) dissolved in DMF (5 mL), triethylamine (1.5 g, 2 cm^3^, 0.14 mmol) and ethyl chloroacetate (2.57 g, 2.25 mL, 21 mmol) were added. The reaction mixture was stirred at room temperature for 24 h. Afterwards, cold water (30 mL) was added, and the precipitate formed was filtered off and recrystallized from propan-2-ol. Yield 80% (4.28 g), white crystals; m.p. 121–122 °C; IR (KBr) ν_max_ (cm^−1^): 1743 (C = O), 3295 (NH); ^1^H NMR (400 MHz, DMSO-*d_6_*): δ 1.19 (t, *J* = 7.2 Hz, 3H, H_14_), 2.77 (t, *J* = 7.2 Hz, 2H, H_8_), 3.26 (q, *J* = 7.2 Hz, 2H, H_7_), 4.03 (s, 2H, H_11_), 4.11 (q, *J* = 7.2 Hz, 2H, H_13_), 5.66 (t, *J* = 6 Hz, 1H, NH), 6.35 (d, 2H, *J* = 7.2 Hz, H_2,6_), 6.50 (t, 1H, *J* = 7.2 Hz, H_4_), 6.35 (d, 2H, *J* = 7.2 Hz, H_3,5_), 7.45–7.51 (m, 2H, H_Ar’_), 7.58–7.64 (m, 3H, H_Ar’_); ^13^C NMR (101 MHz, DMSO-*d_6_*): δ 13.98 (C_14_), 24.64 (C_8_), 33.96 (C_11_), 40.40 (C_7_), 61.24 (C_13_), 111.88, 115.83, 127.36, 128.89, 130.01, 130.10, 132.80, 147.98, 149.11, 154.13 (C_Ar + Ar’_), 168.08 (C_12_); HRMS (ESI): m/z calcd for C_20_H_22_N_4_O_2_S 383.1542 [M]^+^, found 383.1542.

#### 3.1.4. 2-((4-Phenyl-5-(2-(phenylamino)ethyl)-4*H*-1,2,4-triazol-3-yl)thio)acetohydrazide (**3**):

To **2** (2 g, 5 mmol) dissolved in methanol (20 mL), hydrazine hydrate (0.2 g, 0.2 mL, 5 mmol) was added and the reaction mixture was stirred at 60 °C for 24 h. Afterwards, H_2_O (40 mL) was added and the mixture was kept at 4 °C for 24 h. The precipitate was filtered off and recrystallized from ethanol. Yield 94% (1.73 g), white crystals; m.p. 112–113 °C. IR (KBr) ν_max_ (cm^−1^): 1606 (C = O), 3056, 3232, 3331 (NH); ^1^H NMR (400 MHz, DMSO-*d_6_*): δ 2.76 (t, *J* = 7.2 Hz, 2H, H_8_), 3.25 (q, *J* = 7.2 Hz, 2H, H_7_), 3.83 (s, 2H, H_11_), 4.24, 4.29 (2s, 2H, NH_2_), 5.65 (t, *J* = 6 Hz, 1H, NH), 6.34 (d, 2H, *J* = 7.2 Hz, 2H, H_2,6_), 6.49 (d, 1H, *J* = 7.2 Hz, 2H, H_4_), 6.99 (t, 2H, *J* = 7.2 Hz, 2H, H_3,5_), 7.42–7.50 (m, 2H, H_Ar‘_), 7.55–7.63 (m, 3H, H_Ar‘_), 9.32 (s, 1H, *NH*NH_2_); ^13^C NMR (101 MHz, DMSO-*d_6_*): δ 24.66 (C_8_), 34.25 (C_11_), 40.43 (C_7_), 111.88, 115.84, 127.44, 128.91, 129.97, 130.06, 132.89, 147.98, 149.61, 154.02 (C_Ar + Ar’_), 166.08 (C_12_); HRMS (ESI): m/z calcd for C_18_H_20_N_6_OS 369.1498 [M + H]^+^, found 369.1493.

#### 3.1.5. General Procedure for the Synthesis of Compounds **4**–**8**

To hydrazide **3** (0.3 g, 0.8 mmol) dissolved in methanol (10 mL), the corresponding isatin (0.81 mmol) dissolved in methanol (5 mL) was added. The reaction mixture was heated at reflux for 10–75 min until a precipitate formed. The precipitate was filtered off while hot and recrystallized from the DMF/H_2_O mixture.

##### *N*′-(2-oxoindolin-3-ylidene)-2-((4-phenyl-5-(2-(phenylamino)ethyl)-4*H*-1,2,4-triazol-3-yl)thio)acetohydrazide (**4**)

Prepared from isatin; yield 75% (0.3 g), yellow crystals; m.p. 252–253 °C. IR (KBr) ν_max_ (cm^−1^): 1688, 1726 (C = O), 3056, 3182, 3315 (NH); ^1^H NMR (400 MHz, DMSO-*d_6_*): δ 2.76 (t, *J* = 7.2 Hz, 2H, H_8_), 3.16–3.29 (m, 2H, H_7_), 4.10–4.59 (m, 2H, H_11_), 5.65 (s, 1H, NH), 6.33 (d, 2H, *J* = 7.6 Hz, H_2,6_), 6.49 (t, 1H, *J* = 7.6 Hz, 2H, H_4_), 6.91 (d, 1H, *J* = 7.6 Hz, H_Isatin_), 6.98 (t, 2H, *J* = 7.6 Hz, H_3,5_), 7.04–7.15 (m, 1H, H_Ar’_), 7.35–7.56 (m, 3H, H_Isatin_), 7.58–7.63 (m, 3H, H_Ar’_), 7.83–8.60 (m, 1H, H_Ar’_), 10.83 (s, 0.8H, NH_isatin_), 11.30 (s, 0.2H, NH_isatin_), 11.52 (s, 0.8H, NHC_12_), 12.64, 13.41 (2s, 0.2H, NHC_12_); ^13^C NMR (101 MHz, DMSO-*d_6_*): δ 24.70 (C_8_), 34.69 (C_11_), 40.40 (C_7_), 110.66, 111.24, 111.90, 115.14, 115.85, 121.80, 122.70, 126.42, 127.44, 128.92, 130.03, 130.14, 132.88, 142.57, 143.87, 147.98, 154.31 (C_Ar_), 164.43, 171.32 (C_12_,_14_); HRMS (ESI): m/z calcd for C_26_H_23_N_7_O_2_S 498.1713 [M + H]^+^, found 498.1712.

##### *N*′-(5-methoxy-2-oxoindolin-3-ylidene)-2-((4-phenyl-5-(2-(phenylamino)ethyl)-4*H*-1,2,4-triazol-3-yl)thio)acetohydrazide (**5**)

Prepared from 5-methoxyisatin; yield 72% (0.3 g), brown crystals; m.p. 238–239 °C. IR (KBr) ν_max_ (cm^−1^): 1687, 1710 (C = O), 3052, 3182, 3351 (NH); ^1^H NMR (400 MHz, DMSO-*d_6_*): δ 2.74 (t, *J* = 7.2 Hz, 2H, H_8_), 3.23 (s, 2H, H_7_), 3.76 (s, 3H, CH_3_O), 4.15 (s, 1H, H_11_), 4.54 (s, 1H, H_11_), 5.64 (s, 1H, NH), 6.32 (d, 2H, *J* = 7.6 Hz, H_2,6_), 6.48 (t, 1H, *J* = 7.6 Hz, 2H, H_4_), 6.86 (d, 1H, *J* = 7.6 Hz, H_Isatin_), 6.90–7.04 (m, 3H, H_3,5 + Isatin_), 7.06–7.17 (m, 1H, H_Isatin_), 7.46–7.55 (m, 2H, H_Ar‘_), 7.57–7.66 (m, 3H, H_Ar’_), 11.11 (s, 1H, NH_isatin_), 12.68 (2s, 0.6H, NHC_12_), 13.44 (s, 0.4H, NHC_12_); ^13^C NMR (101 MHz, DMSO-*d_6_*): δ 24.66 (C_8_), 33.46, 34.49 (C_11_), 40.39 (C_7_), 55.65 (CH_3_O), 105.77, 111.88, 112.05, 115.84, 117.96, 120.29, 127.43, 128.91, 130.02, 130.13, 132.87, 136.21, 147.97, 154.18, 155.39 (C_Ar_), 162.58, 169.41 (C_12_,_14_); HRMS (ESI): m/z calcd for C_27_H_25_N_7_O_3_S 528.1819 [M + H]^+^, found 528.1817.

##### *N*′-(5-nitro-2-oxoindolin-3-ylidene)-2-((4-phenyl-5-(2-(phenylamino)ethyl)-4*H*-1,2,4-triazol-3-yl)thio)acetohydrazide (**6**)

Prepared from 5-nitroisatin; yield 69% (0.3 g), yellow crystals; m.p. 274–275 °C. IR (KBr) ν_max_ (cm^−1^): 1603, 1701 (C = O), 3052, 3196, 3409 (NH); ^1^H NMR (400 MHz, DMSO-*d_6_*): δ 2.74 (t, *J* = 7.2 Hz, 2H, H_8_), 3.17–3.24 (m, 2H, H_7_), 4.40 (s, 0.8H, H_11_), 4.58 (s, 1.2H, H_11_), 5.64 (s, 1H, NH), 6.32 (d, 2H, *J* = 7.6 Hz, H_2,6_), 6.48 (t, 1H, *J* = 7.6 Hz, 2H, H_4_), 6.98 (t, 2H, *J* = 7.6 Hz, H_3,5_), 7.05–7.18 (m, 1H, H_Ar’_), 7.43–7.54 (m, 2H, H_Isatin + Ar‘_), 7.56–7.68 (m, 3H, H_Ar’_), 8.13–8.46 (m, 2H, H_Isatin_), 11.52 (s, 0.2H, NH_isatin_), 11.91 (s, 0.8H, NH_isatin_), 12.44 (s, 0.6H, NHC_12_), 13.25 (s, 0.4H, NHC_12_); ^13^C NMR (101 MHz, DMSO-*d_6_*): δ 24.66 (C_8_), 33.38 (C_11_), 40.40 (C_7_), 111.48, 111.88, 115.84, 127.44, 127.53, 128.91, 130.03, 130.14, 132.81, 142.83, 147.69, 147.97, 154.28, 149.11 (C_Ar_), 162.79, 169.09 (C_12_,_14_); HRMS (ESI): m/z calcd for C_26_H_22_N_8_O_4_S 543.1564 [M + H]^+^, found 543.1817.

##### *N*′-(5-fluoro-2-oxoindolin-3-ylidene)-2-((4-phenyl-5-(2-(phenylamino)ethyl)-4*H*-1,2,4-triazol-3-yl)thio)acetohydrazide (**7**)

Prepared from 5-fluoroisatin; yield 73% (0.3 g), yellow crystals; m.p. 266–267 °C. IR (KBr) ν_max_ (cm^−1^): 1686, 1713 (C = O), 3053, 3125, 3178 (NH); ^1^H NMR (400 MHz, DMSO-*d_6_*): δ ^1^H NMR (400 MHz, DMSO-*d_6_*): δ 2.75 (t, *J* = 7.2 Hz, 2H, H_8_), 3.24 (q, 2H, *J* = 7.2 Hz, H_7_), 4.15– 4.56 (m, 2H, H_11_), 5.65 (s, 1H, NH), 6.32 (d, 2H, *J* = 7.6 Hz, H_2,6_), 6.49 (t, 1H, *J* = 7.6 Hz, 2H, H_4_), 6.85–6.91 (m, 1H, H_Isatin_), 6.98 (t, 2H, *J* = 7.6 Hz, H_3,5_), 7.20–7.30 (m, 1H, H_Ar’_), 7.35–7.42 (m, 0.4H, H_Isatin_), 7.44–7.57 (m, 2H, H_Ar‘_), 7.58–7.64 (m, 3H, H_Ar’ + Isatin_), 8.12–8.19 (m, 0.6H, H_Isatin_), 10.83 (s, 0.6H, NH_isatin_), 11.31 (s, 0.4H, NH_isatin_), 11.59 (s, 0.6H, NHC_12_), 12.61 (s, 0.2H, NHC_12_), 13.41 (s, 0.2H, NHC_12_); ^13^C NMR (101 MHz, DMSO-*d_6_*): δ 24.66 (C_8_), 34.67 (C_11_), 40.37 (C_7_), 111.88, 115.84, 127.43, 127.51, 128.91, 130.00, 130.02, 130.12, 132.82, 138.80, 140.16, 147.97, 154.28, 156.38, 157.15, 158.73, 159.52 (C_Ar_), 163.11, 169.34 (C_12_,_14_); HRMS (ESI): m/z calcd for C_26_H_22_FN_7_O_2_S 516.1619 [M + H]^+^, found 516.1616.

##### *N*′-(2-oxo-5-(trifluoromethoxy)indolin-3-ylidene)-2-((4-phenyl-5-(2-(phenylamino)ethyl)-4*H*-1,2,4-triazol-3-yl)thio)acetohydrazide (**8**)

Prepared from 5-(trifluoromethoxy)isatin; yield 63% (0.29 g), yellow crystals; m.p. 258–259 °C. IR (KBr) ν_max_ (cm^−1^): 1695, 1719 (C = O), 3059, 3221, 3389 (NH); ^1^H NMR (400 MHz, DMSO-*d_6_*): δ 2.74 (t, *J* = 7.2 Hz, 2H, H_8_), 3.13–3.29 (m, 2H, H_7_), 4.18 (s, 0.8H, H_11_), 4.55 (s, 1.2H, H_11_), 5.64 (s, 1H, NH), 6.32 (d, 2H, *J* = 7.6 Hz, H_2,6_), 6.48 (t, 1H, *J* = 7.6 Hz, 2H, H_4_), 6.98 (t, 2H, *J* = 7.6 Hz, H_3,5_), 7.01–7.08 (m, 1H, H_Ar’_), 7.30–7.40 (m, 1H, H_Isatin_), 7.44–7.60 (m, 6H, H_Ar’ + Isatin_), 11.46 (s, 1H, NH_isatin_), 12.56 (s, 0.6H, NHC_12_), 13.36 (s, 0.4H, NHC_12_); ^13^C NMR (101 MHz, DMSO-*d_6_*): δ 24.65 (C_8_), 33.37 (C_11_), 40.39 (C_7_), 111.87, 112.36, 115.83, 124.68, 127.43, 128.90, 130.01, 130.05, 141.49, 143.62, 147.97, 154.25 (C_Ar_), 162.57, 173.96 (C_12_,_14_); HRMS (ESI): m/z calcd for C_27_H_22_F_3_N_7_O_3_S 582.1536 [M + H]^+^, found 582.1535.

#### 3.1.6. General Procedure for the Synthesis of Compounds **9**–**19**

To hydrazide **3** (1 mmol) dissolved in methanol (10 mL), the corresponding aldehyde (1.1 mmol) dissolved in methanol (5 mL) was added. The reaction mixture was heated at reflux for 30 min–16 h. The reaction mixture was cooled down to 3–4 °C, the precipitate formed was filtered off, washed with methanol, and recrystallized from the DMF/H_2_O mixture.

##### *N*′-(4-methylbenzylidene)-2-((4-phenyl-5-(2-(phenylamino)ethyl)-4*H*-1,2,4-triazol-3-yl)thio)acetohydrazide (**9**)

Prepared from 4-methylbenzaldehyde; yield 64% (0.3 g), white crystals; m.p. 189–190 °C. IR (KBr) ν_max_ (cm^−1^): 1662 (C = O), 3183, 3343 (NH); ^1^H NMR (400 MHz, DMSO-*d_6_*): δ 2.34 (s, 3H, CH_3_), 2.71–2.81 (m, 2H, H_8_), 3.21–3.30 (m, 2H, H_7_), 4.02 (s, 0.8H, H_11_), 4.41 (s, 1.2H, H_11_), 5.66 (t, *J* = 6 Hz, 1H, NH), 6.35 (d, 2H, *J* = 7.2 Hz, 2H, H_2,6_), 6.50 (t, 1H, *J* = 7.2 Hz, 2H, H_4_), 7.00 (t, 2H, *J* = 7.2 Hz, 2H, H_3,5_), 7.26 (d, 1H, *J* = 7.2 Hz, 2H, H_Ar‘_), 7.46–7.52 (m, 2H, H_Ar‘_), 7.53–7.63 (m, 5H, H_Ar’,Ar’’_), 7.97 (s, 0.6H, H_13_), 8.16 (s, 0.4H, H_13_), 11.58 (s, 0.6H, NH), 11.69 (s, 0.4H, NH); ^13^C NMR (101 MHz, DMSO-*d_6_*): δ 21.03 (CH_3_), 24.67 (C_8_), 34.85 (C_11_), 40.42 (C_7_), 111.88, 115.82, 126.82, 127.09, 127.45, 128.90, 129.42, 129.95, 129.99, 131.22, 131.33, 132.99, 139.79, 143.84, 147.04, 147.98, 149.51, 149.69, 154.00, 154.07, 163.24 (C_Ar,Ar’,Ar’’_+ C_13_), 168.43 (C_12_); HRMS (ESI): m/z calcd for C_26_H_26_N_6_OS 471.1968 [M + H]^+^, found 471.1966.

##### *N*′-(4-(dimethylamino)benzylidene)-2-((4-phenyl-5-(2-(phenylamino)ethyl)-4*H*-1,2,4-triazol-3-yl)thio)acetohydrazide (**10**)

Prepared from 4-(dimethylamino)benzaldehyde; yield 54% (0.27 g), grey crystals; m.p. 169–170 °C. IR (KBr) ν_max_ (cm^−1^): 1656 (C = O), 3189, 3323 (NH); ^1^H NMR (400 MHz, DMSO-*d_6_*): δ 2.71–2.80 (m, 2H, H_8_), 2.95, 2.96 (2s, 6H, 2CH_3_), 3.18–3.30 (m, 2H, H_7_), 3.99 (s, 0.8H, H_11_), 4.38 (s, 1.2H, H_11_), 5.65 (s, 1H, NH), 6.34 (d, 2H, *J* = 7.6 Hz, 2H, H_2,6_), 6.50 (t, 1H, *J* = 7.6 Hz, 2H, H_4_), 6.99 (t, 2H, *J* = 7.6 Hz, 2H, H_3,5_), 7.40–7.53 (m, 4H, H_Ar‘_), 7.54–7.67 (m, 3H, H_Ar’,Ar’’_), 7.87 (s, 0.6H, H_13_), 8.03 (s, 0.4H, H_13_), 11.36 (s, 0.6H, NH), 11.44 (s, 0.4H, NH); ^13^C NMR (101 MHz, DMSO-*d_6_*): δ 24.68 (C_8_), 34.98 (C_11_), 35.05 (CH_3_), 40.43 (C_7_), 111.76, 111.88, 115.83, 121.25, 127.43, 127.46, 128.13, 128.45, 128.90, 129.94; 130.00, 132.87, 133.01, 144.59, 147.83, 147.99, 149.59, 149.83, 151.40, 151.54, 153.97, 162.66 (C_Ar,Ar’,Ar’’_+ C_13_), 167.93 (C_12_); HRMS (ESI): m/z calcd for C_27_H_29_N_7_OS 500.2233 [M + H]^+^, found 500.2232.

##### 2-((4-Phenyl-5-(2-(phenylamino)ethyl)-4*H*-1,2,4-triazol-3-yl)thio)-*N*′-(pyridin-4-ylmethylene)acetohydrazide (**11**)

Prepared from 4-pyridinecarboxaldehyde; yield 33% (0.15 g), white crystals; m.p. 209–210 °C. IR (KBr) ν_max_ (cm^−1^): 1682 (C = O), 3179, 3356 (NH); ^1^H NMR (400 MHz, DMSO-*d_6_*): δ 2.70–2.80 (m, 2H, H_8_), 3.18–3.29 (m, 2H, H_7_), 4.03 (s, 0.7H, H_11_), 4.42 (s, 1.3H, H_11_), 5.61 (s, 1H, NH), 6.32 (d, 2H, *J* = 7.6 Hz, 2H, H_2,6_), 6.49 (t, 1H, *J* = 7.6 Hz, 2H, H_4_), 6.98 (t, 2H, *J* = 7.6 Hz, 2H, H_3,5_), 7.41–7.49 (m, 2H, H_Ar‘_), 7.54–7.66 (m, 5H, H_Ar_, _Ar’, Pyridin_), 7.98 (s, 0.7H, H_13_), 8.18 (s, 0.3H, H_13_), 8.62 (2H), 11.88 (s, 0.7H, NH), 12.03 (s, 0.3H, NH); ^13^C NMR (101 MHz, DMSO-*d_6_*): δ 24.82 (C_8_), 34.78 (C_11_), 40.60 (C_7_), 112.10, 116.13, 121.03, 121.26, 127.60, 129.13, 130.19, 130.23, 130.30, 133.05, 141.31, 141.58, 144.92, 148.10, 149.69, 149.92, 150.38, 154.33, 164.22 (C_Ar,Ar’,Ar’’_+ C_13_), 169.23 (C_12_); HRMS (ESI): m/z calcd for C_24_H_23_N_7_OS 458.1764 [M + H]^+^, found 458.1765.

##### 2-((4-Phenyl-5-(2-(phenylamino)ethyl)-4*H*-1,2,4-triazol-3-yl)thio)-*N*′-(pyridin-3-ylmethylene)acetohydrazide (**12**)

Prepared from 3-pyridinecarboxaldehyde; yield 29% (0.13 g), white crystals; m.p. 92–93 °C. IR (KBr) ν_max_ (cm^−1^): 1683 (C = O), 3197, 3393 (NH); ^1^H NMR (400 MHz, DMSO-*d_6_*): δ 2.68–2.81 (m, 2H, H_8_), 3.25 (t, *J* = 7.2 Hz, 2H, H_7_), 4.05 (s, 0.8H, H_11_), 4.23 (s, 1.2H, H_11_), 5.63 (s, 1H, NH), 6.34 (d, 2H, *J* = 7.6 Hz, 2H, H_2,6_), 6.49 (t, 1H, *J* = 7.6 Hz, 2H, H_4_), 6.99 (t, 2H, *J* = 7.6 Hz, 2H, H_3,5_), 7.43–7.52 (m, 3H, H_Ar‘, Pyridin_), 7.54–7.65 (m, 3H, H_Ar’, Pyridin_), 8.04 (s, 0.7H, H_13_), 8.05–8.13 (m, 1H, H_Pyridin_), 8.26 (s, 0.3H, H_13_), 8.52–8.66 (m, 1H, H_Pyridin_), 8.77–8.89 (m, 1H, H_Pyridin_), 11.80 (s, 0.6H, NH), 11.92 (s, 0.4H, NH); ^13^C NMR (101 MHz, DMSO-*d_6_*): δ 24.69 (C_8_), 34.74 (C_11_), 40.44 (C_7_), 111.90, 115.86, 123.94, 127.46, 128.92, 129.97, 130.02, 132.98, 133.39, 140.95, 144.35, 147.99, 148.47, 148.78, 149.61, 150.55, 154.06, 154.12, 163.63 (C_Ar,Ar’,Pyridin_+ C_13_), 168.79 (C_12_); HRMS (ESI): m/z calcd for C_24_H_23_N_7_OS 458.1764 [M + H]^+^, found 458.1760.

##### 4-((2-(2-((4-Phenyl-5-(2-(phenylamino)ethyl)-4*H*-1,2,4-triazol-3-yl)thio)acetyl)hydrazono)methyl)benzoic acid (**13**)

Prepared from 4-formylbenzoic acid; yield 50% (0.25 g), light yellow crystals; m.p. 233–234 °C. IR (KBr) ν_max_ (cm^−1^): 1668, 1708 (C = O), 3174, 3347 (NH); ^1^H NMR (400 MHz, DMSO-*d_6_*): δ 2.70–2.82 (m, 2H, H_8_), 3.25 (t, 2H, *J* = 7.6 Hz, H_7_), 4.05 (s, 0.6H, H_11_), 4.44 (s, 1.4H, H_11_), 5.66 (s, 1H, NH), 6.34 (d, 2H, *J* = 7.6 Hz, H_2,6_), 6.49 (t, 1H, *J* = 7.6 Hz, H_4_), 6.99 (t, 2H, *J* = 7.6 Hz, H_3,5_), 7.42–7.53 (m, 2H, H_Ar‘_), 7.56–7.65 (m, 3H, H_Ar’, Ar’’_), 7.75–7.84 (m, 2H, H_Ar’’_), 8.00 (d, 2H, *J* = 7.6 Hz, 2H, H_Ar“_), 8.06 (s, 0.7H, H_13_), 8.25 (s, 0.3H, H_13_), 11.80 (s, 0.6H, NH), 11.92 (s, 0.4H, NH), 13.14 (s, 1H, OH); ^13^C NMR (101 MHz, DMSO-*d_6_*): δ 24.69 (C_8_), 34.78, 34.97 (C_11_), 40.44 (C_7_), 111.91, 115.86, 126.89 127.15, 127.47, 128.92, 129.78, 129.98, 130.01, 130.11, 131.63, 131.77, 132.85, 132.98, 137.96, 138.13, 142.70, 145.81, 147.99, 149.50, 149.65, 154.05, 154.12, 166.90 (C_Ar,Ar’,Ar’’_+ C_13_), 168.78 (C_12_); HRMS (ESI): m/z calcd for C_26_H_24_N_6_O_3_S 501.1710 [M + H]^+^, found 501.1707.

##### *N*′-((1*H*-pyrrol-2-yl)methylene)-2-((4-phenyl-5-(2-(phenylamino)ethyl)-4*H*-1,2,4-triazol-3-yl)thio)acetohydrazide (**14**)

Prepared from pyrrole-2-carboxaldehyde; yield 65% (0.29 g), white crystals; m.p. 215–216 °C. IR (KBr) ν_max_ (cm^−1^): 1674 (C = O), 3043, 3170, 3327 (NH); ^1^H NMR (400 MHz, DMSO-*d_6_*): δ 2.71–2.81 (m, 2H, H_8_), 3.20–3.32 (m, 2H, H_7_), 4.01 (s, 0.8H, H_11_), 4.38 (s, 1.2H, H_11_), 5.65 (s, 1H, NH), 6.12 (s, 1H, CH_pyrrole_), 6.34 (d, 2H, *J* = 7.6 Hz, 2H, H_2,6_), 6.41–6.53 (m, 2H, H_4_ + CH_pyrrole_), 6.87–6.95 (m, 1H, CH_pyrrole_), 6.99 (t, 2H, *J* = 7.6 Hz, 2H, H_3,5_), 7.44–7.52 (m, 2H, H_Ar‘_), 7.59–7.66 (m, 3H, H_Ar’_), 7.84 (s, 0.6H, H_13_), 8.03 (s, 0.4H, H_13_), 11.31 (s, 0.6H, NH), 11.38 (s, 0.6H, NH), 11.42 (s, 0.4H, NH), 11.50 (s, 0.4H, NH); ^13^C NMR (101 MHz, DMSO-*d_6_*): δ 24.72 (C_8_), 34.85, 35.16 (C_11_), 40.47 (C_7_), 109.22, 109.29, 111.91, 112.58, 113.50, 115.86, 122.03, 122.61, 126.72, 126.96, 127.50, 128.92, 129.94, 130.00, 132.88, 133.05, 136.56, 140.18, 147.99, 149.62, 149.74, 153.98, 154.08, 162.62 (C_Ar,Ar’,Pyrrole_+ C_13_), 167.92 (C_12_); HRMS (ESI): m/z calcd for C_23_H_23_N_7_OS 446.1764 [M + H]^+^, found 446.1762.

##### *N*′-((3-phenyl-1*H*-pyrazol-4-yl)methylene)-2-((4-phenyl-5-(2-(phenylamino)ethyl)-4*H*-1,2,4-triazol-3-yl)thio)acetohydrazide (**15**)

Prepared from 3-phenyl-1*H*-pyrazole-4-carboxaldehyde; yield 89% (0.46 g), white crystals; m.p. 119–120 °C. IR (KBr) ν_max_ (cm^−1^): 1678 (C = O), 3056, 3185, 3314 (NH); ^1^H NMR (400 MHz, DMSO-*d_6_*): δ 2.71–2.79 (m, 2H, H_8_), 3.21–3.29 (m, 2H, H_7_), 3.96 (s, 0.8H, H_11_), 4.30, 4.32 (2s, 1.2H, H_11_), 5.65 (t, 1H, *J* = 6 Hz, NH), 6.34 (d, 2H, *J* = 7.6 Hz, 2H, H_2,6_), 6.49 (t, 1H, *J* = 7.6 Hz, H_4_), 6.99 (t, 2H, *J* = 7.6 Hz, 2H, H_3,5_), 7.34–7.48 (m, 4H, H_Ar‘ + Ar“_), 7.52–7.67 (m, 6H, H_Ar’ + Ar“_), 7.88–8.25 (m, 2H, CH_pyrazole +_ H_13_)), 11.31 (s, 0.6H, NH), 11.50 (s, 0.4H, NH), 13.35 (s, 0.4H, NH_pyrazole_), 13.45, 13.48 (2s, 0.6H, NH_pyrazole_), ^13^C NMR (101 MHz, DMSO-*d_6_*): δ 24.70 (C_8_), 34.82, 35.01 (C_11_), 40.43 (C_7_), 111.89, 113.58, 115.84, 127.44, 127.48, 128.22, 128.45, 128.91, 129.00, 129.95, 129.99, 130.02, 130.08, 132.86, 133.03, 147.99, 149.51, 149.66, 154.01, 154.08, 162.65 (C_Ar,Ar’, Ar“, Pyrazole_+ C_13_), 167.83 (C_12_); HRMS (ESI): m/z calcd for C_28_H_26_N_8_OS 523.2029 [M + H]^+^, found 523.2027.

##### *N*′-((1-methyl-1*H*-pyrazol-3-yl)methylene)-2-((4-phenyl-5-(2-(phenylamino)ethyl)-4*H*-1,2,4-triazol-3-yl)thio)acetohydrazide (**16**)

Prepared from 1-methyl-1*H*-pyrazole-3-carbaldehyde; yield 98% (0.45 g), light brown crystals; m.p. 62–63 °C. IR (KBr) ν_max_ (cm^−1^): 1688 (C = O), 3195, 3339 (NH); ^1^H NMR (400 MHz, DMSO-*d_6_*): δ 2.70–2.81 (m, 2H, H_8_), 3.19–3.31 (m, 2H, H_7_), 3.40 (s, 3H, CH_3_), 3.86 (s, 0.8H, H_11_), 4.35, 4.43 (2s, 1.2H, H_11_), 5.65 (s, 1H, NH), 6.34 (d, 2H, *J* = 7.6 Hz, 2H, H_2,6_), 6.49 (t, 1H, *J* = 7.6 Hz, H_4_), 6.52–6.65 (m, 1H, CH_pyrazole_), 6.99 (t, 2H, *J* = 7.6 Hz, 2H, H_3,5_), 7.35–8.18 (m, 7H, H_Ar‘ +_ CH_pyrazole_ + H_13_)), 11.53 (s, 0.4H, NH), 11.67 (s, 0.3H, NH), 12.06 (s, 0.2H, NH), 12.75, (s, 0.1H, NH); ^13^C NMR (101 MHz, DMSO-*d_6_*): δ 24.69 (C_8_), 34.69, 35.00 (C_11_), 38.78 (CH_3_), 40.45 (C_7_), 103.03, 103.13,108.61, 111.93, 115.88, 127.45, 127.49, 128.94, 129.98, 130.03, 130.12, 131.95, 132.51, 132.64, 132.86, 133.01, 138.73, 141.76, 145.12, 146.91, 147.03, 149.54, 149.69, 154.05, 154.13, 163.19 (C_Ar,Ar’, Pyrazole_+ C_13_), 168.28 (C_12_); HRMS (ESI): m/z calcd for C_23_H_24_N_8_OS 461.1873 [M + H]^+^, found 461.1875.

##### *N*′-(2-hydroxybenzylidene)-2-((4-phenyl-5-(2-(phenylamino)ethyl)-4*H*-1,2,4-triazol-3-yl)thio)acetohydrazide (**17**)

Prepared from 2-hydroxybenzaldehyde; yield 64% (0.3 g), white crystals; m.p. 209–210 °C. IR (KBr) ν_max_ (cm^−1^): 1663 (C = O), 3200, 3323 (NH); ^1^H NMR (400 MHz, DMSO-*d_6_*): δ 2.71–2.80 (m, 2H, H_8_), 3.21–3.30 (m, 2H, H_7_), 4.03 (s, 1H, H_11_), 4.41 (s, 1H, H_11_), 5.65 (t, 1H, *J* = 7.6 Hz, NH), 6.34 (d, 2H, *J* = 7.6 Hz, H_2,6_), 6.49 (t, 1H, *J* = 7.6 Hz, 2H, H_4_), 6.84–6.93 (m, 2H, H_Ar’’_), 6.99 (t, 2H, *J* = 7.6 Hz, H_3,5_), 7.21–7.32 (m, 1H, H_Ar’’_), 7.45–7.51 (m, 2H, H_Ar‘_), 7.54–7.67 (m, 4H, H_Ar’,Ar’’_), 8.31 (s, 0.45H, H_13_), 8.42 (s, 0.55H, H_13_), 10.06 (s, 0.4H, OH), 10.99 (s, 0.6H, OH), 11.59 (s, 0.4H, NH), 11.96 (s, 0.6H, NH); ^13^C NMR (101 MHz, DMSO-*d_6_*): δ 24.67 (C_8_), 34.65, 34.93 (C_11_), 40.42 (C_7_), 111.89, 115.84, 116.16, 116.36, 118.61, 119.37, 120.01, 126.24, 127.42, 127.46, 128.91, 129.18, 129.96, 130.00, 130.11, 131.25, 131.49, 132.83, 132.98, 141.27, 147.19, 147.99, 149.45, 149.71, 154.14, 156.42, 157.28, 163.28 (C_Ar,Ar’,Ar’’_+ C_13_), 168.23 (C_12_); HRMS (ESI): m/z calcd for C_25_H_24_N_6_O_2_S 473.1760 [M + H]^+^, found 473.1760.

##### *N*′-(2-hydroxy-5-nitrobenzylidene)-2-((4-phenyl-5-(2-(phenylamino)ethyl)-4*H*-1,2,4-triazol-3-yl)thio)acetohydrazide (**18**)

Prepared from 2-hydroxy-5-nitrobenzaldehyde; yield 67% (0.35 g), yellow crystals; m.p. 219–220 °C. IR (KBr) ν_max_ (cm^−1^): 1687 (C = O), 3182, 3416 (NH); ^1^H NMR (400 MHz, DMSO-*d_6_*): δ 2.71–2.81 (m, 2H, H_8_), 3.20–3.30 (m, 2H, H_7_), 4.05 (s, 1H, H_11_), 4.45 (s, 1H, H_11_), 5.67 (s, 1H, NH), 6.34 (d, 2H, *J* = 7.6 Hz, H_2,6_), 6.49 (t, 1H, *J* = 7.6 Hz, 2H, H_4_), 6.98 (t, 2H, *J* = 7.6 Hz, H_3,5_), 7.07–7.11 (m, 1H, H_Ar’’_), 7.46–7.53 (m, 2H, H_Ar‘_), 7.56–7.63 (m, 3H, H_Ar’_), 8.11–8.19 (m, 1H, H_Ar’’_), 8.30 (s, 0.5H, H_13_), 8.55 (s, 0.5H, H_13_), 8.50, 8.51 (2s, 1H, H_Ar’’_), 11.77 (s, 1H, NH), 12.14 (s, 1H, OH); ^13^C NMR (101 MHz, DMSO-*d_6_*): δ 24.68 (C_8_), 34.69, 34.79 (C_11_), 40.43 (C_7_), 111.91, 115.85, 116.73, 117.08, 119.85, 121.01, 121.41, 123.65, 126.52, 126.67, 127.46, 128.91, 129.97, 130.13, 132.83, 132.98, 138.36, 139.93, 140.01, 143.50, 147.98, 149.44, 149.65, 154.06, 154.16, 161.90, 162.47, 163.69 (C_Ar,Ar’,Ar’’_+ C_13_), 168.58 (C_12_); HRMS (ESI): m/z calcd for C_25_H_23_N_7_O_4_S 518.1611 [M + H]^+^, found 518.1609.

##### *N*′-(4-(methylthio)benzylidene)-2-((4-phenyl-5-(2-(phenylamino)ethyl)-4*H*-1,2,4-triazol-3-yl)thio)acetohydrazide (**19**)

Prepared from 4-(methylthio)benzaldehyde; yield 74% (0.3 g), white crystals; m.p. 196–197 °C. IR (KBr) ν_max_ (cm^−1^): 1677 (C = O), 3180, 3316 (NH); ^1^H NMR (400 MHz, DMSO-*d_6_*): δ 2.70–2.81 (m, 2H, H_8_), 3.18–3.30 (m, 2H, H_7_), 3.36 (s, 3H, CH_3_), 4.02 (s, 0.8H, H_11_), 4.41 (s, 1.2H, H_11_), 5.65 (t, *J* = 6 Hz, 1H, NH), 6.34 (d, 2H, *J* = 7.2 Hz, 2H, H_2,6_), 6.49 (t, 1H, *J* = 7.2 Hz, 2H, H_4_), 6.99 (t, 2H, *J* = 7.2 Hz, 2H, H_3,5_), 7.30 (d, 1H, *J* = 7.2 Hz, 2H, H_Ar‘_), 7.43–7.51 (m, 2H, H_Ar‘_), 7.55–7.66 (m, 5H, H_Ar’,Ar’’_), 7.96 (s, 0.6H, H_13_), 8.14 (s, 0.4H, H_13_), 11.61 (s, 0.6H, NH), 11.71 (s, 0.4H, NH); ^13^C NMR (101 MHz, DMSO-*d_6_*): δ 14.28 (CH_3_), 24.68 (C_8_), 34.87, 34.97 (C_11_), 40.44 (C_7_), 111.90, 115.85, 125.59, 125.64, 127.27, 127.43, 127.46, 127.53, 128.92, 129.97, 130.01, 130.04,130.10, 130.37, 130.45, 132.85, 132.99, 140.88, 141.11, 143.40, 146.63, 147.99, 149.54, 149.72, 154.02, 154.09, 163.28 (C_Ar,Ar’,Ar’’_ + C_13_), 168.45 (C_12_); HRMS (ESI): m/z calcd for C_26_H_26_N_6_OS_2_ 503.1689 [M + H]^+^, found 503.1691.

### 3.2. Pharmacology

#### 3.2.1. Cell Culturing

The human malignant melanoma cell line IGR39, human triple-negative breast cancer MDA-MB-231, and human pancreatic carcinoma cell line Panc-1 were obtained from the American Type Culture Collection (ATCC, Manassas, VA, USA). Human foreskin fibroblasts (HF) CRL-4001 were originally obtained from ATCC and kindly provided by Prof. Helder Santos (University of Helsinki, Finland). IGR39, MDA-MB-231, Panc-1, and HF were cultured in Dulbecco’s Modified Eagle’s GlutaMAX medium (Gibco (Carlsbad, CA, USA)). The medium was supplemented with 10,000 U/mL penicillin, 10 mg/mL streptomycin (Gibco), and 10% fetal bovine serum (Gibco). Cell cultures were grown at 37°C in a humidified atmosphere containing 5% CO_2_. They were used until the passage of 20.

#### 3.2.2. Cell Viability Assay

The effect of synthesized compounds on cell viability was studied using 3-(4,5-dimethylthiazol-2-yl)-2,5-diphenyltetrazolium bromide (MTT; Sigma-Aldrich Co., St Louis, MO, USA) assay, as described elsewhere [73]. Briefly, IGR39, MDA-MB-231, Panc-1, and HF cells were seeded in 96-well plates (Corning) in triplicate at a volume of 100 μL (IGR39, MDA-MB-231 and Panc-1: 4 × 10^3^ cells/well; HF: 5 × 10^3^ cells/well). After 24 h, cells were treated with 50 μM of tested compounds. After 72 h, the MTT reagent was added and cells were incubated for 4 h. Then the medium was aspirated, and the formed formazan crystals were dissolved in 100 μL DMSO (Sigma-Aldrich Co., St. Louis, MO, USA). The absorbance was measured at 570 and 630 nm using a multi-detection microplate reader. The compound effect on cell viability was calculated using the formula:

(1) $$Relative\;cell\;viability\;\left(\%\right)=\frac{A-A_{0}}{A_{NC}-A_{0}}$$

where

*A*—*mean of absorbance of the tested compound*,
*A_0_*—*mean of absorbance of blank (no cells, positive control)*, and
*A_NC_*—*mean of absorbance of negative control (only cells, no treatment)*.

The EC_50_ values of the most active hydrazones **4**, **7**, **8**, **10**, **14**, **17**, and **18** were established using the same MTT procedure. The compound serial dilutions from 50 µM to 1.56 µM were made in a medium and added to the cells in triplicates. The EC_50_ value representing the concentration of a compound causing 50% reduction of cancer cell metabolic activity was calculated using the Hill equation.

#### 3.2.3. Wound Healing’ Assay

The ‘Wound healing’ assay was used to evaluate the inhibitory effect of the most active hydrazones **4**, **7**, **8**, **10**, **14**, **17**, and **18** on cell migration, as described elsewhere [74]. Briefly, melanoma IGR39, human triple-negative breast cancer MDA-MB-231, and pancreatic carcinoma Panc-1 cells were seeded in 24-well plates at a density of 6 × 10^4^ cells/well and incubated for 48 h in previously described cell culture medium at 37°C in a humidified atmosphere containing 5% CO_2_. Then the scratch was made using a 100 µL pipette tip. Cells were washed once with PBS, and the fresh medium containing 10 µM of compounds **4**, **7**, **8**, **10**, **14**, **17**, and **18** was added. Medium containing 0.1% DMSO was used as a negative control. Cells were incubated at 37 °C in a humidified atmosphere containing 5% CO_2_. 

The ‘Wounds’ were captured at the intervals of 0 h, 24 h, 48 h and 72 h (only for the Panc-1 cell line) from scratch under the phase contrast microscope at a 4× magnification. The wound area was analysed using the ImageJ program (National Institute of Health, Bethesda, MD, USA).

#### 3.2.4. Compound Activity in Cell 3D Cultures (Spheroids)

Cancer cell spheroids were formed using the magnetic 3D Bioprinting method, as described elsewhere [75]. Briefly, melanoma IGR39, triple-negative breast cancer MDA-MB-231 cells, pancreatic cancer Panc-1 cells, and human fibroblasts at 70% confluency in a 6-well plate were incubated with Nanoshuttle (n3D Biosciences, Inc., Houston, TX, USA) for 8 h at 37°C in a humidified atmosphere containing 5% CO_2_. Then cells were trypsinized, centrifuged, and seeded into an ultra-low attachment 96-well plate in a volume of 100 µL (1.5 × 10^3^ cancer cells and 1.5 × 10^3^ human fibroblasts/well). The plate was placed on a magnetic drive and incubated for 2 days at 37°C in a humidified atmosphere containing 5% CO_2_. Then the fresh medium containing 10 µM of the tested compound was added to the wells. Spheroids were captured every two days using the Olympus IX73 inverted microscope (OLYMPUS CORPORATION, Tokyo, Japan). Quantitative analysis of compound anticancer activity in spheroids was performed using ImageJ, version 1.53o (National Institutes of Health, USA) and Microsoft Office Excel 2016 software (Microsoft Corporation, Redmond, WA, USA).

On the last day of incubation, 10 µL of WST-1 reagent (Sigma-Aldrich Co, St. Louis, MO, USA) was added to each well with spheroids. After 10 h of incubation, 50 µL of liquid from each well were transferred to the new 96-well plate and the absorbance was measured at 460 and 530 nm using a multi-detection microplate reader. Spheroid cell viability was calculated using a formula provided in Section 3.2.2. 

#### 3.2.5. Statistical Analysis

All biological experiments were repeated at least three times, calculating the mean and standard deviation. The data was processed using the Microsoft Office Excel 2016 software (Microsoft Corporation, Redmond, WA, USA) and the IBM SPSS Statistics version 27.0 package. Statistical analysis was performed by using the Student’s t-test. The level of significance was set as *p* < 0.05. In order to determine significant differences between values, analysis of variance (ANOVA) followed by a Tukey post-hoc test was performed.

## 4. Conclusions

In conclusion, a series of novel hydrazone derivatives bearing the 2-((3-(2-phenylamino)ethyl)-4-phenyl-4*H*-1,2,4-triazol-5-yl)sulfanyl moiety along with a 5-substituted 2-oxindole fragment and various aromatic and heterocyclic moieties were synthesized and evaluated for their anticancer properties. In the anticancer activity assay, the melanoma IGR39 cell line appeared to be more sensitive to the treatment with the tested hydrazone derivatives, compared to the triple-negative breast cancer MDA-MB-231 and pancreatic carcinoma Panc-1 cell lines. Compounds **4**, **14**, and **18** were the most active among all synthesized compounds in 3D cell cultures. *N*′-(4-(dimethylamino)benzylidene)-2-((4-phenyl-5-(2-(phenylamino)ethyl)-4*H*-1,2,4-triazol-3-yl)thio)acetohydrazide (**10**) inhibited all cancer cell migration and could be further tested as an antimetastatic candidate. 

## Data Availability

Data is contained within the article and Supplementary Materials.

## Supplementary Materials

The following are available online at https://www.mdpi.com/article/10.3390/ph15081026/s1, Figures S1–S57 display 1H NMR, 13C NMR, and HRMS spectra of compounds 2–19; Figures S58–60 provide data on the 10 µM compound effect on cell viability after 24, 48, and 72 h. Procedure S1 describes the MTT assay used for evaluation of 10 µM compound effects on cell viability after 24, 48 and 72 h.

## References

[1] Siegel R.L., Miller K.D., Fuchs H.E., Jemal A. (2021). Cancer Statistics, 2021. CA A Cancer J. Clin..

[2] Pancreatic Cancer: Statistics. https://www.cancer.net/cancer-types/pancreatic-cancer/statistics..

[3] Trapani D., Ginsburg O., Fadelu T., Lin N.U., Hassett M., Ilbawi A.M., Anderson B.O., Curigliano G. (2022). Global Challenges and Policy Solutions in Breast Cancer Control. Cancer Treat. Rev..

[4] Davis L.E., Shalin S.C., Tackett A.J. (2019). Current State of Melanoma Diagnosis and Treatment. Cancer Biol. Ther..

[5] Ghosh S., Ramarao T.A., Samanta P.K., Jha A., Satpati P., Sen A. (2021). Triazole Based Isatin Derivatives as Potential Inhibitor of Key Cancer Promoting Kinases- Insight from Electronic Structure, Docking and Molecular Dynamics Simulations. J. Mol. Graph. Model..

[6] Spivak A.Y., Nedopekina D.A., Gubaidullin R.R., Dubinin M.V., Belosludtsev K.N. (2021). Conjugation of Natural Triterpenic Acids with Delocalized Lipophilic Cations: Selective Targeting Cancer Cell Mitochondria. J. Pers. Med..

[7] Bhullar K.S., Lagarón N.O., McGowan E.M., Parmar I., Jha A., Hubbard B.P., Rupasinghe H.P.V. (2018). Kinase-Targeted Cancer Therapies: Progress, Challenges and Future Directions. Mol. Cancer.

[8] Dhokne P., Sakla A.P., Shankaraiah N. (2021). Structural Insights of Oxindole Based Kinase Inhibitors as Anticancer Agents: Recent Advances. Eur. J. Med. Chem..

[9] Abdelli A., Azzouni S., Plais R., Gaucher A., Efrit M.L., Prim D. (2021). Recent Advances in the Chemistry of 1,2,4-Triazoles: Synthesis, Reactivity and Biological Activities. Tetrahedron Lett..

[10] Grytsai O., Valiashko O., Penco-Campillo M., Dufies M., Hagege A., Demange L., Martial S., Pagès G., Ronco C., Benhida R. (2020). Synthesis and Biological Evaluation of 3-Amino-1,2,4-Triazole Derivatives as Potential Anticancer Compounds. Bioorg. Chem..

[11] Gao M., Diao Q., Gao F., Sun X., Xiao J. (2021). Bis-Triazole-Containing Compounds with Anticancer Potential: A Short Review. Curr. Top. Med. Chem..

[12] Turky A., Bayoumi A.H., Sherbiny F.F., El-Adl K., Abulkhair H.S. (2021). Unravelling the Anticancer Potency of 1,2,4-Triazole-*N*-Arylamide Hybrids through Inhibition of STAT3: Synthesis and *in Silico* Mechanistic Studies. Mol. Divers..

[13] Aggarwal R., Sumran G. (2020). An Insight on Medicinal Attributes of 1,2,4-Triazoles. Eur. J. Med. Chem..

[14] Kaur R., Ranjan Dwivedi A., Kumar B., Kumar V. (2016). Recent Developments on 1,2,4-Triazole Nucleus in Anticancer Compounds: A Review. Anti-Cancer Agents Med. Chem..

[15] Wen X., Zhou Y., Zeng J., Liu X. (2020). Recent Development of 1,2,4-Triazole-Containing Compounds as Anticancer Agents. Curr. Top. Med. Chem..

[16] Shaker R.M. (2006). The Chemistry of Mercapto- and Thione- Substituted 1,2,4-Triazoles and Their Utility in Heterocyclic Synthesis. Arkivoc.

[17] Slivka M.V., Korol N.I., Fizer M.M. (2020). Fused Bicyclic 1,2,4-triazoles with One Extra Sulfur Atom: Synthesis, Properties, and Biological Activity. J. Heterocycl. Chem..

[18] Küçükgüzel Ş.G., Çıkla-Süzgün P. (2015). Recent Advances Bioactive 1,2,4-Triazole-3-Thiones. Eur. J. Med. Chem..

[19] Patel K.R., Brahmbhatt J.G., Pandya P.A., Daraji D.G., Patel H.D., Rawal R.M., Baran S.K. (2021). Design, Synthesis and Biological Evaluation of Novel 5-(4-Chlorophenyl)-4-Phenyl-4*H*-1,2,4-Triazole-3-Thiols as an Anticancer Agent. J. Mol. Struct..

[20] Rollas S., Küçükgüzel S. (2007). Biological Activities of Hydrazone Derivatives. Molecules.

[21] de Oliveira Carneiro Brum J., França T.C.C., LaPlante S.R., Villar J.D.F. (2020). Synthesis and Biological Activity of Hydrazones and Derivatives: A Review. Mini Rev. Med. Chem..

[22] Popiołek Ł. (2021). Updated Information on Antimicrobial Activity of Hydrazide–Hydrazones. Int. J. Mol. Sci..

[23] Demurtas M., Baldisserotto A., Lampronti I., Moi D., Balboni G., Pacifico S., Vertuani S., Manfredini S., Onnis V. (2019). Indole Derivatives as Multifunctional Drugs: Synthesis and Evaluation of Antioxidant, Photoprotective and Antiproliferative Activity of Indole Hydrazones. Bioorg. Chem..

[24] Alam M., Verma G., Shaquiquzzaman M., Marella A., Akhtar M., Ali M. (2014). A Review Exploring Biological Activities of Hydrazones. J. Pharm. Bioall. Sci..

[25] Ferraz de Paiva R.E., Vieira E.G., Rodrigues da Silva D., Wegermann C.A., Costa Ferreira A.M. (2021). Anticancer Compounds Based on Isatin-Derivatives: Strategies to Ameliorate Selectivity and Efficiency. Front. Mol. Biosci..

[26] Rizzo M., Porta C. (2017). Sunitinib in the Treatment of Renal Cell Carcinoma: An Update on Recent Evidence. Ther. Adv. Urol..

[27] Nath R., Pathania S., Grover G., Akhtar M.J. (2020). Isatin Containing Heterocycles for Different Biological Activities: Analysis of Structure Activity Relationship. J. Mol. Struct..

[28] Khetmalis Y.M., Shivani M., Murugesan S., Chandra Sekhar K.V.G. (2021). Oxindole and Its Derivatives: A Review on Recent Progress in Biological Activities. Biomed. Pharmacother..

[29] Cheke R.S., Patil V.M., Firke S.D., Ambhore J.P., Ansari I.A., Patel H.M., Shinde S.D., Pasupuleti V.R., Hassan M.I., Adnan M. (2022). Therapeutic Outcomes of Isatin and Its Derivatives against Multiple Diseases: Recent Developments in Drug Discovery. Pharmaceuticals.

[30] Kaur M., Singh M., Chadha N., Silakari O. (2016). Oxindole: A Chemical Prism Carrying Plethora of Therapeutic Benefits. Eur. J. Med. Chem..

[31] Aneja B., Khan N.S., Khan P., Queen A., Hussain A., Rehman M.T., Alajmi M.F., El-Seedi H.R., Ali S., Hassan M.I. (2019). Design and Development of Isatin-Triazole Hydrazones as Potential Inhibitors of Microtubule Affinity-Regulating Kinase 4 for the Therapeutic Management of Cell Proliferation and Metastasis. Eur. J. Med. Chem..

[32] Tumosienė I., Peleckis A., Jonuškienė I., Vaickelionienė R., Kantminienė K., Šiugždaitė J., Beresnevičius Z.J., Mickevičius V. (2018). Synthesis of Novel 1,2- and 2-Substituted Benzimidazoles with High Antibacterial and Antioxidant Activity. Monatsh. Chem..

[33] Tumosienė I., Kantminienė K., Klevinskas A., Petrikaitė V., Jonuškienė I., Mickevičius V. (2020). Antioxidant and Anticancer Activity of Novel Derivatives of 3-[(4-Methoxyphenyl)Amino]Propanehydrazide. Molecules.

[34] Meleddu R., Petrikaite V., Distinto S., Arridu A., Angius R., Serusi L., Škarnulytė L., Endriulaitytė U., Paškevičiu Tė M., Cottiglia F. (2019). Investigating the Anticancer Activity of Isatin/Dihydropyrazole Hybrids. ACS Med. Chem. Lett..

[35] Tumosienė I., Jonuškienė I., Kantminienė K., Mickevičius V., Petrikaitė V. (2021). Novel N-Substituted Amino Acid Hydrazone-Isatin Derivatives: Synthesis, Antioxidant Activity, and Anticancer Activity in 2D and 3D Models *In Vitro*. Int. J. Mol. Sci..

[36] Onnis V., Cocco M.T., Fadda R., Congiu C. (2009). Synthesis and Evaluation of Anticancer Activity of 2-Arylamino-6-Trifluoromethyl-3-(Hydrazonocarbonyl)Pyridines. Bioorg. Med. Chem..

[37] Easmon J., Pürstinger G., Thies K.-S., Heinisch G., Hofmann J. (2006). Synthesis, Structure−Activity Relationships, and Antitumor Studies of 2-Benzoxazolyl Hydrazones Derived from Alpha-(*N*)-Acyl Heteroaromatics. J. Med. Chem..

[38] Li Petri G., Spanò V., Spatola R., Holl R., Raimondi M.V., Barraja P., Montalbano A. (2020). Bioactive Pyrrole-Based Compounds with Target Selectivity. Eur. J. Med. Chem..

[39] Karrouchi K., Radi S., Ramli Y., Taoufik J., Mabkhot Y., Al-aizari F., Ansar M. (2018). Synthesis and Pharmacological Activities of Pyrazole Derivatives: A Review. Molecules.

[40] Xia Y., Fan C.-D., Zhao B.-X., Zhao J., Shin D.-S., Miao J.-Y. (2008). Synthesis and Structure–Activity Relationships of Novel 1-Arylmethyl-3-Aryl-1*H*-Pyrazole-5-Carbohydrazide Hydrazone Derivatives as Potential Agents against A549 Lung Cancer Cells. Eur. J. Med. Chem..

[41] Zebbiche Z., Tekin S., Küçükbay H., Yüksel F., Boumoud B. (2021). Synthesis and Anticancer Properties of Novel Hydrazone Derivatives Incorporating Pyridine and Isatin Moieties. Arch. Pharm..

[42] Braeuer R.R., Watson I.R., Wu C.-J., Mobley A.K., Kamiya T., Shoshan E., Bar-Eli M. (2014). Why Is Melanoma so Metastatic?. Pigment Cell Melanoma Res..

[43] Manjunath M., Choudhary B. (2021). Triple-Negative Breast Cancer: A Run-through of Features, Classification and Current Therapies. Oncol. Lett..

[44] Hruban R.H., Gaida M.M., Thompson E., Hong S.-M., Noë M., Brosens L.A., Jongepier M., Offerhaus G.J.A., Wood L.D. (2019). Why Is Pancreatic Cancer so Deadly? The Pathologist’s View. J. Pathol..

[45] Jaaks P., Coker E.A., Vis D.J., Edwards O., Carpenter E.F., Leto S.M., Dwane L., Sassi F., Lightfoot H., Barthorpe S. (2022). Effective Drug Combinations in Breast, Colon and Pancreatic Cancer Cells. Nature.

[46] Esteva F.J., Hubbard-Lucey V.M., Tang J., Pusztai L. (2019). Immunotherapy and Targeted Therapy Combinations in Metastatic Breast Cancer. Lancet Oncol..

[47] Tumosiene I., Kantminiene K., Pavilonis A., Mazeliene Z., Beresnevicius Z.J. (2009). Synthesis of Azole Derivatives from 3-Phenylaminopropanhydrazide and Evaluation of Their Antimicrobial Efficacy. Heterocycles.

[48] Tisovský P., Csicsai K., Donovalová J., Šandrik R., Sokolík R., Gáplovský A. (2020). Effect of a =X-NH-Fragment, (X = C, N), on Z/E Isomerization and ON/OFF Functionality of Isatin Arylhydrazones, ((Arylamino)Methylene)Indolin-2-Ones and Their Anions. Molecules.

[49] Strelciunaite V., Jonuskiene I., Anusevicius K., Tumosiene I., Siugzdaite J., Ramanauskaite I., Mickevicius V. (2016). Synthesis of Novel Benzimidazoles 2-Functionalized with Pyrrolidinone and γ-Amino Acid with a High Antibacterial Activity. Heterocycles.

[50] Pal A., Curtin J.F., Kinsella G.K. (2021). *In Silico* and *in Vitro* Screening for Potential Anticancer Candidates Targeting GPR120. Bioorg. Med. Chem. Lett..

[51] Abebe F.A., Hopkins M.D., Vodnala S.N., Sheaff R.J., Lamar A.A. (2021). Development of a Rapid *In Vitro* Screening Assay Using Metabolic Inhibitors to Detect Highly Selective Anticancer Agents. ACS Omega.

[52] Shoemaker R.H. (2006). The NCI60 Human Tumour Cell Line Anticancer Drug Screen. Nat. Rev. Cancer.

[53] Sharma S.V., Haber D.A., Settleman J. (2010). Cell Line-Based Platforms to Evaluate the Therapeutic Efficacy of Candidate Anticancer Agents. Nat. Rev. Cancer.

[54] Kozar I., Margue C., Rothengatter S., Haan C., Kreis S. (2019). Many Ways to Resistance: How Melanoma Cells Evade Targeted Therapies. Biochim. Biophys. Acta (BBA)-Rev. Cancer.

[55] Nedeljković M., Damjanović A. (2019). Mechanisms of Chemotherapy Resistance in Triple-Negative Breast Cancer-How We Can Rise to the Challenge. Cells.

[56] Long J., Zhang Y., Yu X., Yang J., LeBrun D., Chen C., Yao Q., Li M. (2011). Overcoming Drug Resistance in Pancreatic Cancer. Expert Opin. Ther. Targets.

[57] Tiago M., de Oliveira E.M., Brohem C.A., Pennacchi P.C., Paes R.D., Haga R.B., Campa A., de Moraes Barros S.B., Smalley K.S., Maria-Engler S.S. (2014). Fibroblasts Protect Melanoma Cells from the Cytotoxic Effects of Doxorubicin. Tissue Eng. Part A.

[58] Ham I.-H., Wang L., Lee D., Woo J., Kim T.H., Jeong H.Y., Oh H.J., Choi K.S., Kim T.-M., Hur H. (2022). Curcumin Inhibits the Cancer-associated Fibroblast-derived Chemoresistance of Gastric Cancer through the Suppression of the JAK/STAT3 Signaling Pathway. Int. J. Oncol..

[59] Blagosklonny M.V., Pardee A.B. (2001). Exploiting Cancer Cell Cycling for Selective Protection of Normal Cells. Cancer Res..

[60] Skvortsov D.A., Kalinina M.A., Zhirkina I.V., Vasilyeva L.A., Ivanenkov Y.A., Sergiev P.V., Dontsova O.A. (2021). From Toxicity to Selectivity: Coculture of the Fluorescent Tumor and Non-Tumor Lung Cells and High-Throughput Screening of Anticancer Compounds. Front. Pharmacol..

[61] Bedia C., Casas J., Andrieu-Abadie N., Fabriàs G., Levade T. (2011). Acid Ceramidase Expression Modulates the Sensitivity of A375 Melanoma Cells to Dacarbazine. J. Biol. Chem..

[62] Caporali S., Alvino E., Lacal P.M., Levati L., Giurato G., Memoli D., Caprini E., Antonini Cappellini G.C., D’Atri S. (2016). Targeting the PI3K/AKT/MTOR Pathway Overcomes the Stimulating Effect of Dabrafenib on the Invasive Behavior of Melanoma Cells with Acquired Resistance to the BRAF Inhibitor. Int. J. Oncol..

[63] Eddy K., Shah R., Chen S. (2021). Decoding Melanoma Development and Progression: Identification of Therapeutic Vulnerabilities. Front. Oncol..

[64] Garcia E., Luna I., Persad K.L., Agopsowicz K., Jay D.A., West F.G., Hitt M.M., Persad S. (2021). Inhibition of Triple Negative Breast Cancer Metastasis and Invasiveness by Novel Drugs That Target Epithelial to Mesenchymal Transition. Sci. Rep..

[65] Malakouti P., Mohammadi M., Boshagh M.A., Amini A., Rezaee M.A., Rahmani M.R. (2022). Combined Effects of Pioglitazone and Doxorubicin on Migration and Invasion of MDA-MB-231 Breast Cancer Cells. J. Egypt. Nat. Canc. Inst..

[66] Mishra R., Yuan L., Patel H., Karve A.S., Zhu H., White A., Alanazi S., Desai P., Merino E.J., Garrett J.T. (2021). Phosphoinositide 3-Kinase (PI3K) Reactive Oxygen Species (ROS)-Activated Prodrug in Combination with Anthracycline Impairs PI3K Signaling, Increases DNA Damage Response and Reduces Breast Cancer Cell Growth. Int. J. Mol. Sci..

[67] Ahn K., Moon O Y., Ji Y.G., Cho H.J., Lee D.H. (2018). Synergistic Anti-Cancer Effects of AKT and SRC Inhibition in Human Pancreatic Cancer Cells. Yonsei Med. J..

[68] Barbosa M.A.G., Xavier C.P.R., Pereira R.F., Petrikaitė V., Vasconcelos M.H. (2021). 3D Cell Culture Models as Recapitulators of the Tumor Microenvironment for the Screening of Anti-Cancer Drugs. Cancers.

[69] Zanoni M., Piccinini F., Arienti C., Zamagni A., Santi S., Polico R., Bevilacqua A., Tesei A. (2016). 3D Tumor Spheroid Models for *in Vitro* Therapeutic Screening: A Systematic Approach to Enhance the Biological Relevance of Data Obtained. Sci. Rep..

[70] Golas J.M., Lucas J., Etienne C., Golas J., Discafani C., Sridharan L., Boghaert E., Arndt K., Ye F., Boschelli D.H. (2005). SKI-606, a Src/Abl Inhibitor with *in Vivo* Activity in Colon Tumor Xenograft Models. Cancer Res..

[71] Aihara A., Iwawaki T., Abe-Fukasawa N., Otsuka K., Saruhashi K., Mikashima T., Nishino T. (2022). Small Molecule LATS Kinase Inhibitors Block the Hippo Signaling Pathway and Promote Cell Growth under 3D Culture Conditions. J. Biol. Chem..

[72] Gandalovičová A., Rosel D., Fernandes M., Veselý P., Heneberg P., Čermák V., Petruželka L., Kumar S., Sanz-Moreno V., Brábek J. (2017). Migrastatics—Anti-Metastatic and Anti-Invasion Drugs: Promises and Challenges. Trends Cancer.

[73] Čeponytė U., Paškevičiūtė M., Petrikaitė V. (2018). Comparison of NSAIDs Activity in COX-2 Expressing and Non-Expressing 2D and 3D Pancreatic Cancer Cell Cultures. Cancer Manag. Res..

[74] Stravinskiene D., Sliziene A., Baranauskiene L., Petrikaite V., Zvirbliene A. (2020). Inhibitory Monoclonal Antibodies and Their Recombinant Derivatives Targeting Surface-Exposed Carbonic Anhydrase XII on Cancer Cells. Int. J. Mol. Sci..

[75] Bytautaite M., Petrikaite V. (2020). Comparative Study of Lipophilic Statin Activity in 2D and 3D *in Vitro* Models of Human Breast Cancer Cell Lines MDA-MB-231 and MCF-7. Onco Targets Ther..

