# Supplementary material for: The Effect of 1,2,4-Triazole-3-thiol Derivatives Bearing Hydrazone Moiety on Cancer Cell Migration and Growth of Melanoma, Breast, and Pancreatic Cancer Spheroids
Source: Pharmaceuticals (Basel). 2022 Aug 20;15(8):1026. doi: 10.3390/ph15081026 (PMC9416745; doi:10.3390/ph15081026)
Supplement: Supplementary file 1 [file pharmaceuticals-15-01026-s001.zip › pharmaceuticals-1841560-supplementary.pdf]

Supplementary Material

The effect of 1,2,4-triazole-3-thiol derivatives bearing hydrazone moiety on cancer cell migration and growth of melanoma, breast and pancreatic cancer spheroids

Aida Šermukšnytė[1], Kristina Kantminienė[2]*, Ilona Jonuškienė[1], Ingrida Tumosienė[1], and Vilma Petrikaitė[3]*

[1] Department of Organic Chemistry, Kaunas University of Technology, Radvilėnų pl. 19, 50254 Kaunas, Lithuania
[2] Department of Physical and Inorganic Chemistry, Kaunas University of Technology, Radvilėnų pl. 19, 50254 Kaunas, Lithuania
[3] Laboratory of Drug Targets Histopathology, Institute of Cardiology, Lithuanian University of Health Sciences, Sukilėlių pr. 13, 50162 Kaunas, Lithuania
* Correspondence: kristina.kantminiene@ktu.lt (K.K.); vilma.petrikaite@lsmuni.lt (V.P.)

Table of contents

Figure S1. ^{1}H NMR (400 MHz, DMSO-d$_6$) spectrum of **1**

Figure S2. ^{13}C NMR (101 MHz, DMSO-d$_6$) spectrum of **1**

Figure S3. HRMS spectrum of **1**

Figure S4. ^{1}H NMR (400 MHz, DMSO-d$_6$) spectrum of **2**

Figure S5. ^{13}C NMR (101 MHz, DMSO-d$_6$) spectrum of **2**

Figure S6. HRMS spectrum of **2**

Figure S7. ^{1}H NMR (400 MHz, DMSO-d$_6$) spectrum of **3**

Figure S8. ^{13}C NMR (101 MHz, DMSO-d$_6$) spectrum of **3**

Figure S9. HRMS spectrum of **3**

Figure S10. ^{1}H NMR (400 MHz, DMSO-d$_6$) spectrum of **4**

Figure S11. ^{13}C NMR (101 MHz, DMSO-d$_6$) spectrum of **4**

Figure S12. HRMS spectrum of **4**

Figure S13. ^{1}H NMR (400 MHz, DMSO-d$_6$) spectrum of **5**

Figure S14. ^{13}C NMR (101 MHz, DMSO-d$_6$) spectrum of **5**

Figure S15. HRMS spectrum of **5**

Figure S16. ^{1}H NMR (400 MHz, DMSO-d$_6$) spectrum of **6**

Figure S17. ^{13}C NMR (400 MHz, DMSO-d$_6$) spectrum of **6**

Figure S18. HRMS spectrum of **6**

Figure S19. ^{1}H NMR (400 MHz, DMSO-d_6) spectrum of **7**

Figure S20. ^{13}C NMR (101 MHz, DMSO-d_6) spectrum of **7**

Figure S21. HRMS spectrum of **7**

Figure S22. ^{1}H NMR (400 MHz, DMSO-d_6) spectrum of **8**

Figure S23. ^{13}C NMR (101 MHz, DMSO-d_6) spectrum of **8**

Figure S24. HRMS spectrum of **8**

Figure S25. ^{1}H NMR (400 MHz, DMSO-d_6) spectrum of **9**

Figure S26. ^{13}C NMR (101 MHz, DMSO-d_6) spectrum of **9**

Figure S27. HRMS spectrum of **9**

Figure S28. ^{1}H NMR (400 MHz, DMSO-d_6) spectrum of **10**

Figure S29. ^{13}C NMR (101 MHz, DMSO-d_6) spectrum of **10**

Figure S30. HRMS spectrum of **10**

Figure S31. ^{1}H NMR (400 MHz, DMSO-d_6) spectrum of **11**

Figure S32. ^{13}C NMR (101 MHz, DMSO-d_6) spectrum of **11**

Figure S33. HRMS spectrum of **11**

Figure S34. ^{1}H NMR (400 MHz, DMSO-d_6) spectrum of **12**

Figure S35. ^{13}C NMR (101 MHz, DMSO-d_6) spectrum of **12**

Figure S36. HRMS spectrum of **12**

Figure S37. ^{1}H NMR (400 MHz, DMSO-d_6) spectrum of **13**

Figure S38. ^{13}C NMR (101 MHz, DMSO-d_6) spectrum of **13**

Figure S39. HRMS spectrum of **13**

Figure S40. ^{1}H NMR (400 MHz, DMSO-d_6) spectrum of **14**

Figure S41. ^{13}C NMR (101 MHz, DMSO-d_6) spectrum of **14**

Figure S42. HRMS spectrum of **14**

Figure S43. ^{1}H NMR (400 MHz, DMSO-d_6) spectrum of **15**

Figure S44. ^{13}C NMR (101 MHz, DMSO-d_6) spectrum of **15**

Figure S45. HRMS spectrum of **15**

Figure S46. ^{1}H NMR (400 MHz, DMSO-d_6) spectrum of **16**

Figure S47. ^{13}C NMR (101 MHz, DMSO-d_6) spectrum of **16**

Figure S48. HRMS spectrum of **16**

Figure S49. ^{1}H NMR (400 MHz, DMSO-d_6) spectrum of **17**

Figure S50. ^{13}C NMR (101 MHz, DMSO-d_6) spectrum of **17**

Figure S51. HRMS spectrum of **17**

Figure S52. ^{1}H NMR (400 MHz, DMSO-d_6) spectrum of **18**

Figure S53. ^{13}C NMR (101 MHz, DMSO-d_6) spectrum of **18**

Figure S54. HRMS spectrum of **18**

Figure S55. ^{1}H NMR (400 MHz, DMSO-d_6) spectrum of **19**

Figure S56. ^{13}C NMR (101 MHz, DMSO-d_6) spectrum of **19**

Figure S57. HRMS spectrum of **19**

Figure S58. The effect of 10 μM compound concentration on MDA-MB-231 cell line viability after 24 and 48 h.

Figure S59. The effect of 10 μM compound concentration on IGR39 cell viability after 24 h and 48 h.

Figure S60. The effect of 10 μM compound concentration on Panc-1 cell line viability after 24, 48, and 72 h.

Procedure S1. Evaluation of 10 μM compound concentration effect on cell line viability.

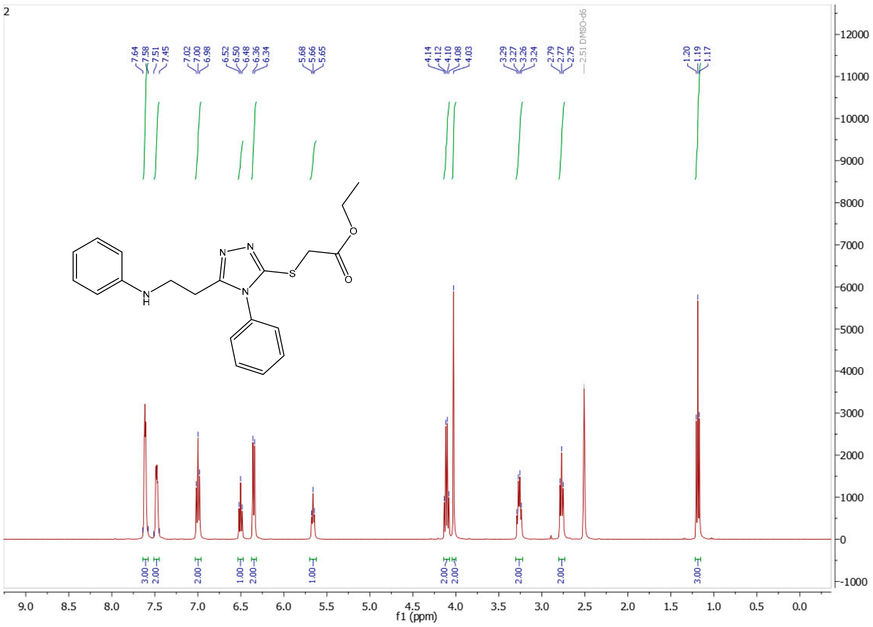

Figure S1. ^{1}H NMR (400 MHz, DMSO-d6) spectrum of **2**

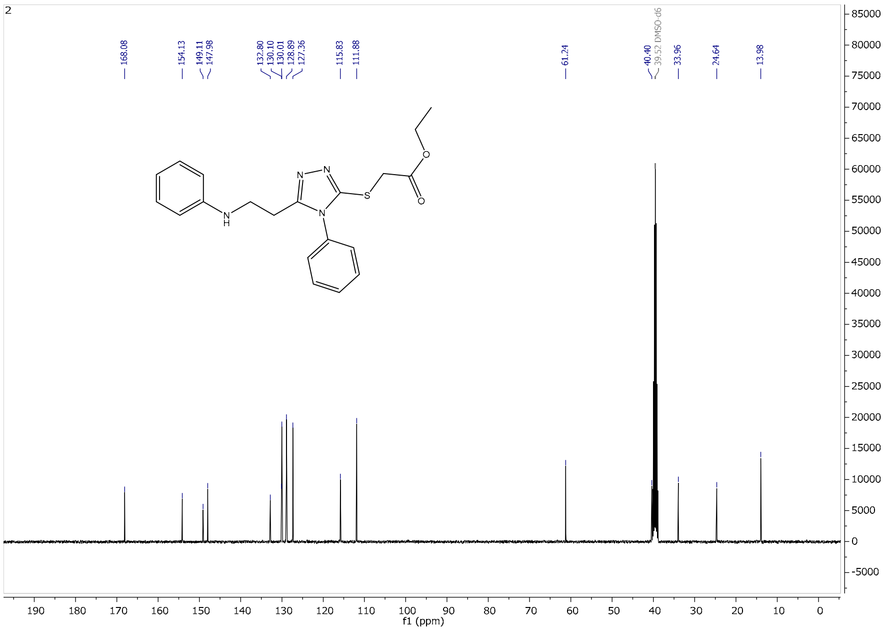

Figure S2. ^{13}C NMR (101 MHz, DMSO-d6) spectrum of **2**

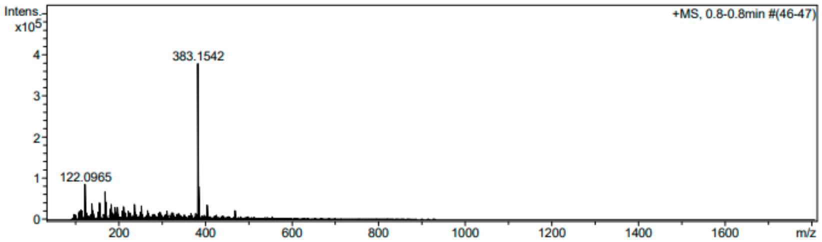

Meas. m/z	#	Formula	Score	m/z	err [ppm]	Mean err [ppm]	mSigma	rdb	e⁻ Conf	N-Rule
383.1542	1	C 19 H 27 O 6 S	47.96	383.1523	-4.9	-5.0	12.1	6.5	even	ok
	2	C 20 H 23 N 4 O 2 S	100.00	383.1536	-1.4	-1.5	16.3	11.5	even	ok
	3	C 16 H 23 N 4 O 7	36.75	383.1561	5.1	6.0	22.3	7.5	even	ok

Figure S3. HRMS spectrum of **2**

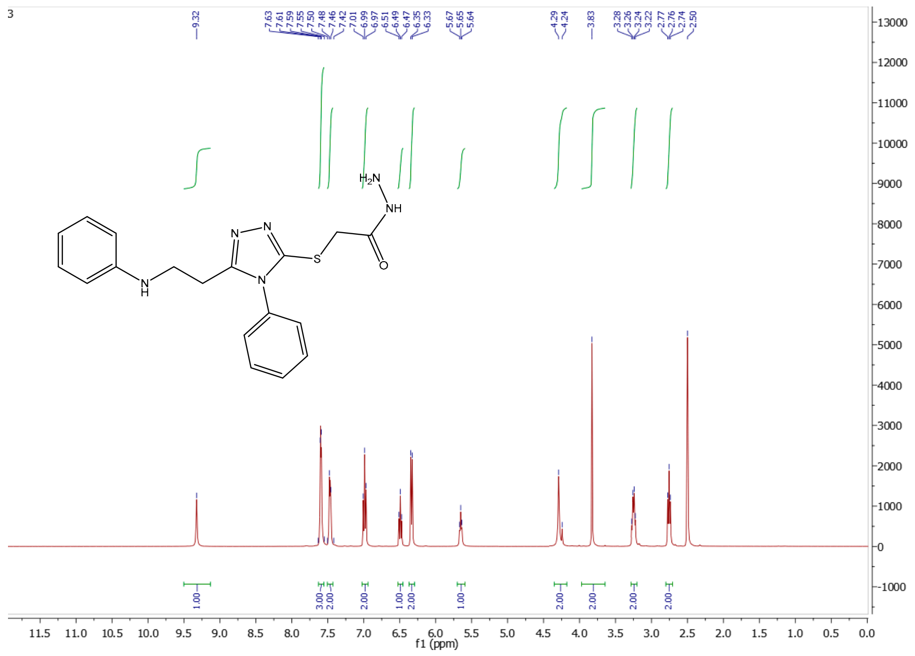

Figure S7. ^{1}H NMR (400 MHz, DMSO-d_6) spectrum of **3**

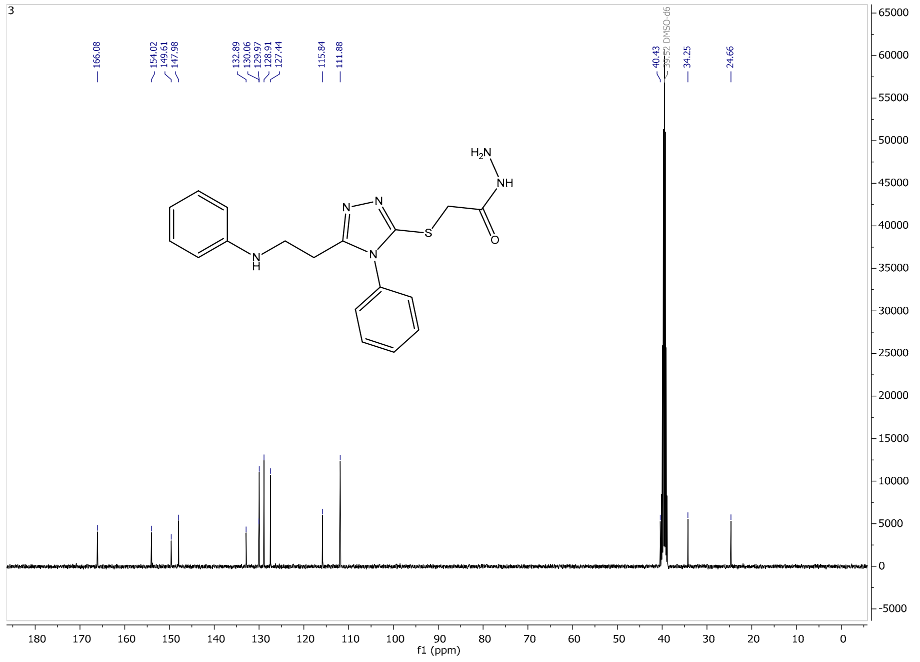

Figure S8. ^{13}C NMR (101 MHz, DMSO-d_6) spectrum of **3**

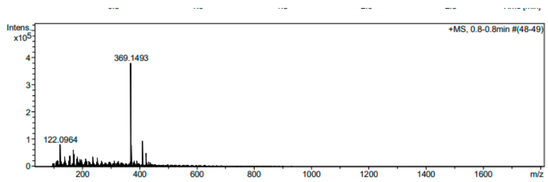

Meas. m/z	#	Formula	Score	m/z	err [ppm]	Mean err [ppm]	mSigma	rdb	e⁻ Conf	N-Rule
369.1493	1	C 18 H 21 N 6 O S	100.00	369.1492	-0.1	-0.1	11.7	11.5	even	ok
	2	C 17 H 25 N 2 O 5 S	48.32	369.1479	-3.8	-3.6	13.2	6.5	even	ok
	3	C 20 H 26 Na O 3 S	88.68	369.1495	0.6	0.8	14.1	7.5	even	ok
	4	C 13 H 18 N 10 Na O 2	36.26	369.1506	3.7	5.0	26.8	9.5	even	ok
	5	C 25 H 21 O 3	43.47	369.1485	-2.0	-0.7	34.8	15.5	even	ok
	6	C 13 H 25 N 2 O 10	34.18	369.1504	3.0	4.1	36.0	2.5	even	ok

Figure S9. HRMS spectrum of **3**

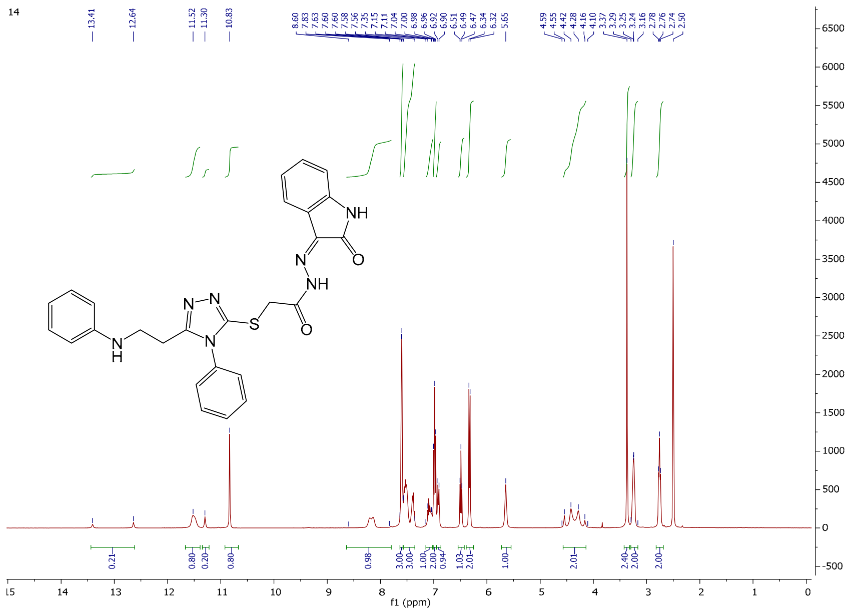

Figure S10. ^{1}H NMR (400 MHz, DMSO-d_6) spectrum of **4**

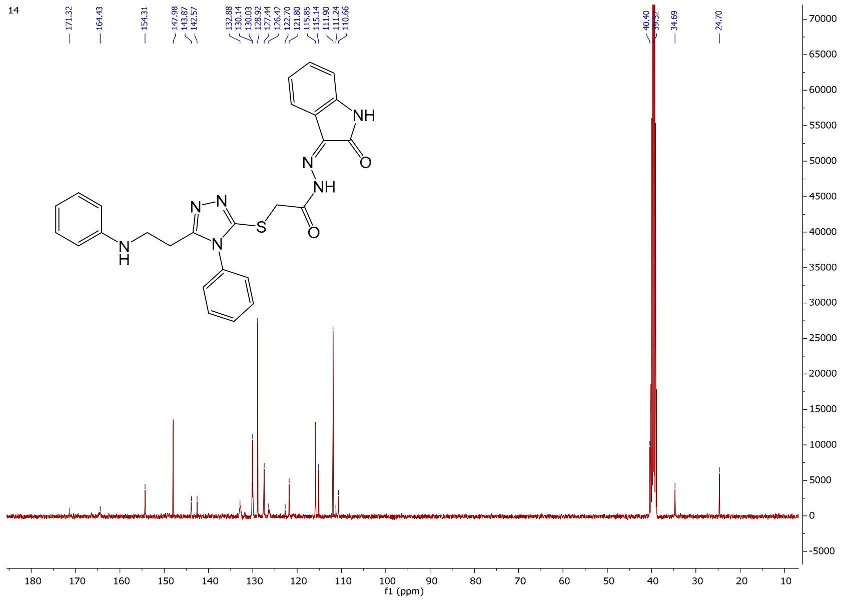

Figure S11. ^{13}C NMR (101 MHz, DMSO-d_6) spectrum of **4**

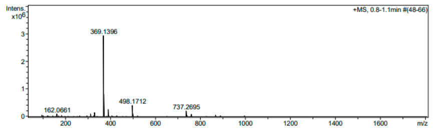

Meas. m/z	#	Formula	Score	m/z	err [ppm]	Mean err [ppm]	mSigma	rdb	e⁻ Conf	N-Rule
369.1396	1	C 27 H 17 N 2	100.00	369.1386	-2.6	-1.7	24.3	20.5	even	ok
	2	C 19 H 21 N 4 O 2 S	61.15	369.1380	-4.4	-3.7	28.0	11.5	even	ok
498.1712	1	C 22 H 24 N 7 O 7	49.20	498.1732	3.9	4.8	12.6	14.5	even	ok
	2	C 25 H 28 N 3 O 6 S	46.45	498.1693	-3.8	-4.0	17.7	13.5	even	ok
	3	C 19 H 16 N 17 O	100.00	498.1718	1.2	2.0	19.1	20.5	even	ok
	4	C 21 H 28 N 3 O 11	98.69	498.1718	1.2	2.3	19.4	9.5	even	ok
	5	C 26 H 24 N 7 O 2 S	90.21	498.1707	-1.1	-1.3	24.9	18.5	even	ok
	6	C 33 H 24 N O 4	40.19	498.1700	-2.5	-1.4	41.9	22.5	even	ok
	7	C 34 H 20 N 5	49.06	498.1713	0.2	1.3	54.7	27.5	even	ok

Figure S12. HRMS spectrum of **4**

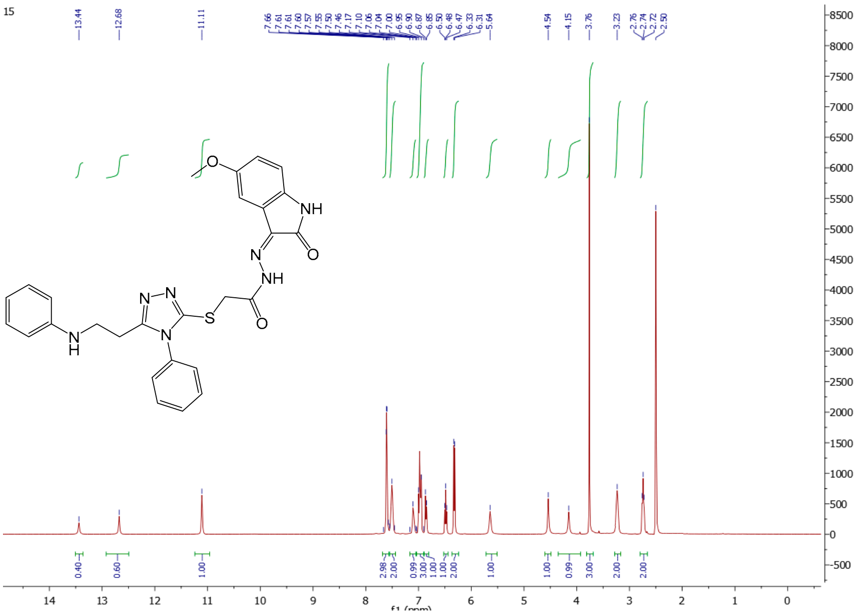

Figure S13. ^{1}H NMR (400 MHz, DMSO-d$_6$) spectrum of **5**

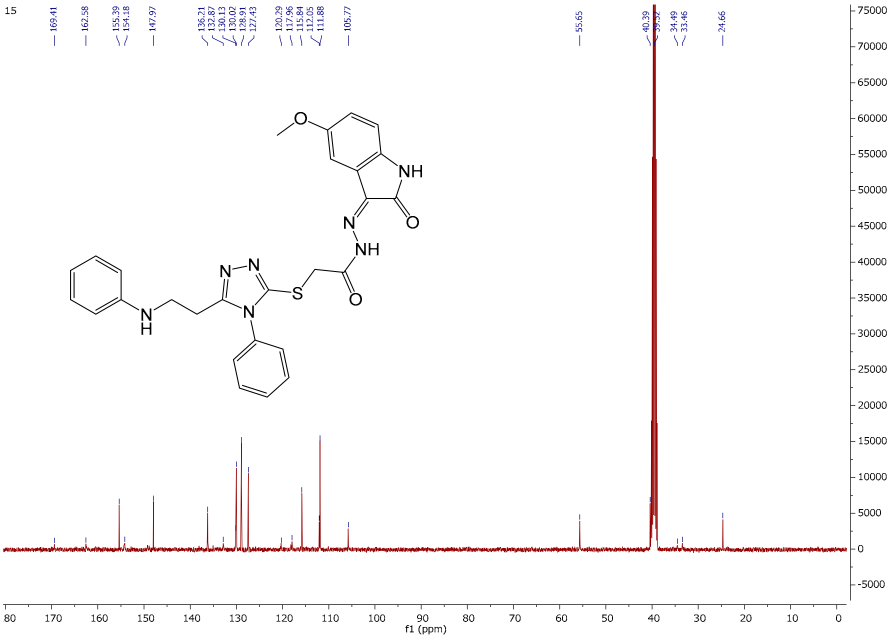

Figure S14. ^{13}C NMR (101 MHz, DMSO-d$_6$) spectrum of **5**

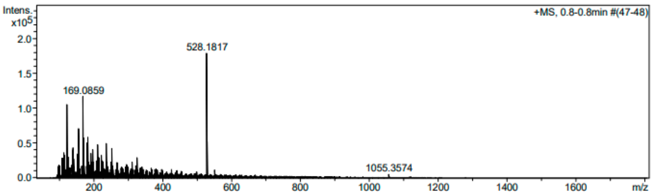

Meas. m/z	#	Formula	Score	m/z	err [ppm]	Mean err [ppm]	mSigma	rdb	e⁻ Conf	N-Rule
528.1817	1	C 26 H 30 N 3 O 7 S	51.56	528.1799	-3.5	-3.5	16.7	13.5	even	ok
	2	C 20 H 18 N 17 O 2	92.92	528.1824	1.3	2.2	22.5	20.5	even	ok
	3	C 22 H 30 N 3 O 12	91.32	528.1824	1.3	2.3	23.1	9.5	even	ok
	4	C 27 H 26 N 7 O 3 S	100.00	528.1812	-0.9	-1.0	23.3	18.5	even	ok
	5	C 34 H 26 N O 5	46.97	528.1805	-2.2	-1.1	38.9	22.5	even	ok
	6	C 35 H 22 N 5 O	54.59	528.1819	0.3	1.4	51.5	27.5	even	ok

Figure S15. HRMS spectrum of **5**

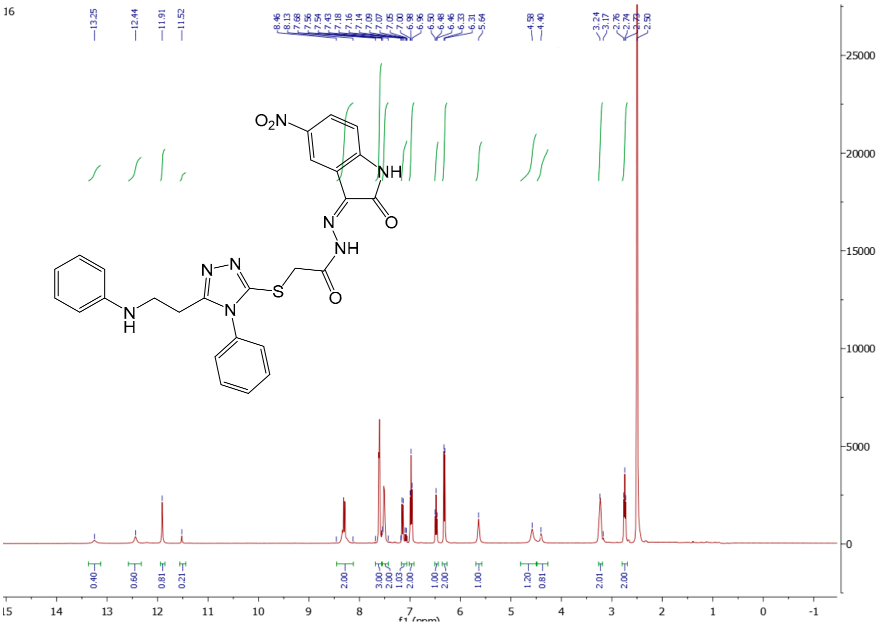

Figure S16. ^{1}H NMR (400 MHz, DMSO-d$_6$) spectrum of **6**

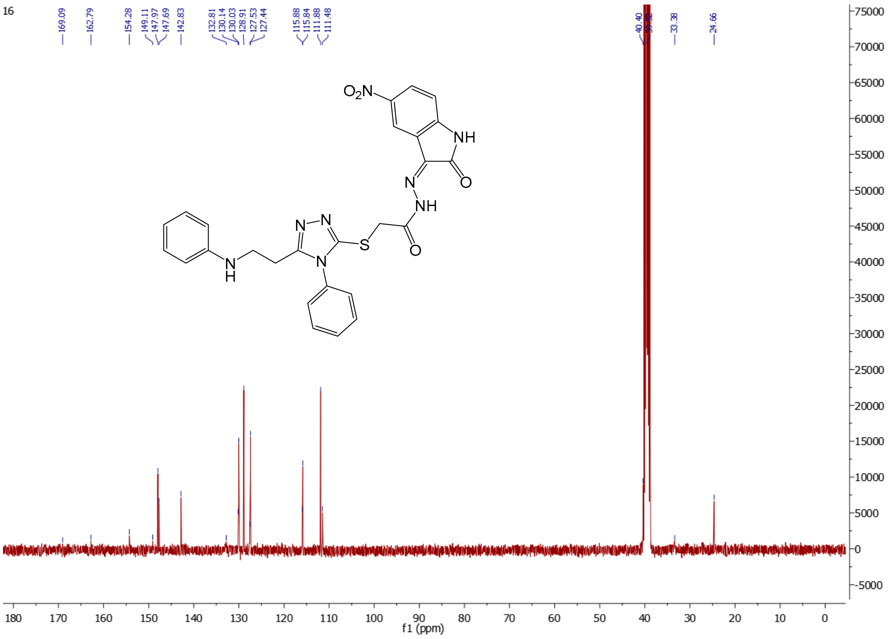

Figure S17. ^{13}C NMR (101 MHz, DMSO-d$_6$) spectrum of **6**

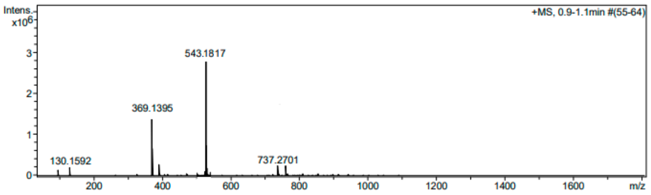

Meas. m/z	#	Formula	Score	m/z	err [ppm]	Mean err [ppm]	mSigma	rdb	e⁻ Conf	N-Rule
369.1395	1	C 19 H 21 N 4 O 2 S	78.61	369.1380	-4.1	-3.7	18.1	11.5	even	ok
	2	C 15 H 21 N 4 O 7	94.90	369.1405	2.7	3.8	23.9	7.5	even	ok
	3	C 14 H 25 O 11	100.00	369.1391	-0.9	0.2	35.6	2.5	even	ok
	4	C 27 H 17 N 2	66.35	369.1386	-2.3	-1.0	41.3	20.5	even	ok
	5	C 22 H 17 N 13 O 3 S	34.79	543.1817	-1.3	-3.3	69.6	23.5	even	ok

Figure S18. HRMS spectrum of **6**

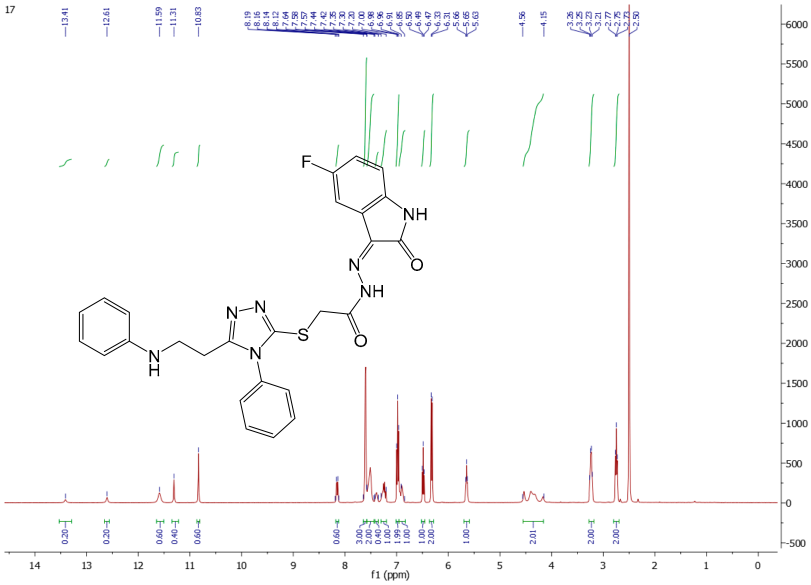

Figure S19. ¹H NMR (400 MHz, DMSO-d6) spectrum of **7**

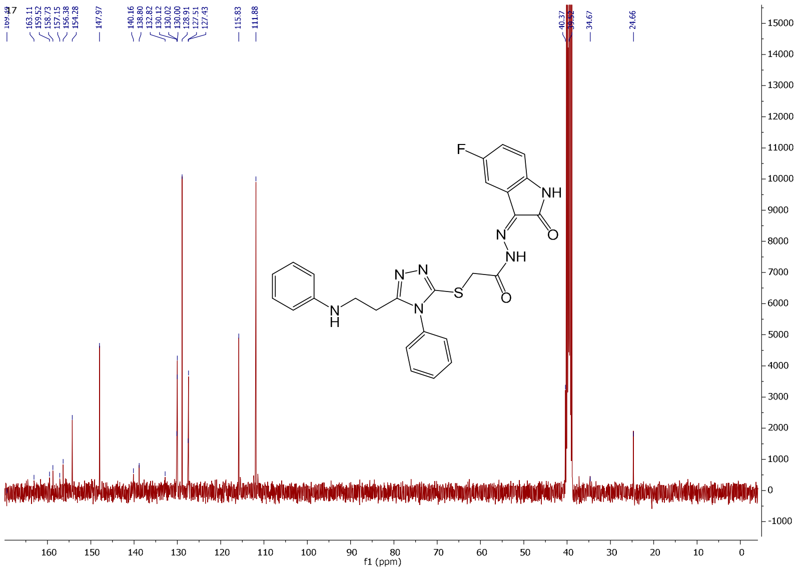

Figure S20. ¹³C NMR (101 MHz, DMSO-d6) spectrum of **7**

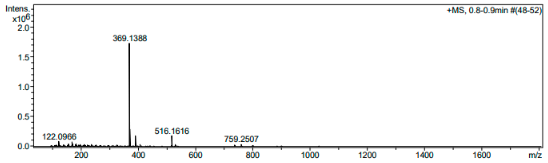

Meas. m/z	#	Formula	Score	m/z	err [ppm]	Mean err [ppm]	mSigma	rdb	e⁻ Conf	N-Rule
369.1388	1	C 15 H 21 N 4 O 7	47.97	369.1405	4.7	5.5	5.6	7.5	even	ok
	2	C 14 H 25 O 11	100.00	369.1391	1.0	1.9	8.0	2.5	even	ok
	3	C 11 H 17 N 10 O 5	69.90	369.1378	-2.6	-1.8	11.1	8.5	even	ok
	4	C 12 H 13 N 14 O	90.14	369.1391	1.0	1.9	13.7	13.5	even	ok
	5	C 15 H 26 F O 7 S	59.12	369.1378	-2.6	-2.5	19.2	2.5	even	ok
	6	C 16 H 22 F N 4 O 3 S	76.67	369.1391	1.0	1.1	21.8	7.5	even	ok
	7	C 19 H 21 N 4 O 2 S	44.88	369.1380	-2.1	-2.0	35.4	11.5	even	ok
	8	C 24 H 18 F N 2 O	21.48	369.1398	2.7	3.8	56.4	16.5	even	ok
	9	C 27 H 17 N 2	24.58	369.1386	-0.4	0.7	65.2	20.5	even	ok
516.1616	1	C 25 H 22 N 7 O 6	75.69	516.1626	1.9	2.8	11.5	18.5	even	ok
	2	C 25 H 27 F N 3 O 6 S	47.27	516.1599	-3.3	-3.5	12.1	13.5	even	ok
	3	C 24 H 26 N 3 O 10	100.00	516.1613	-0.7	0.4	13.9	13.5	even	ok
	4	C 26 H 23 F N 7 O 2 S	94.91	516.1612	-0.7	-1.0	16.0	18.5	even	ok
	5	C 22 H 14 N 17	89.62	516.1613	-0.7	0.1	19.1	24.5	even	ok
	6	C 21 H 18 N 13 O 4	40.35	516.1599	-3.3	-2.5	20.3	19.5	even	ok
	7	C 21 H 27 F N 3 O 11	58.64	516.1624	1.5	2.6	28.4	9.5	even	ok
	8	C 29 H 22 N 7 O S	35.61	516.1601	-2.9	-2.1	31.1	22.5	even	ok
	9	C 33 H 23 F N O 4	43.92	516.1606	-2.1	-0.9	34.1	22.5	even	ok
	10	C 33 H 26 N O 3 S	31.62	516.1628	2.3	3.1	43.8	21.5	even	ok
	11	C 34 H 19 F N 5	46.03	516.1619	0.5	1.7	46.9	27.5	even	ok

Figure S21. HRMS spectrum of **7**

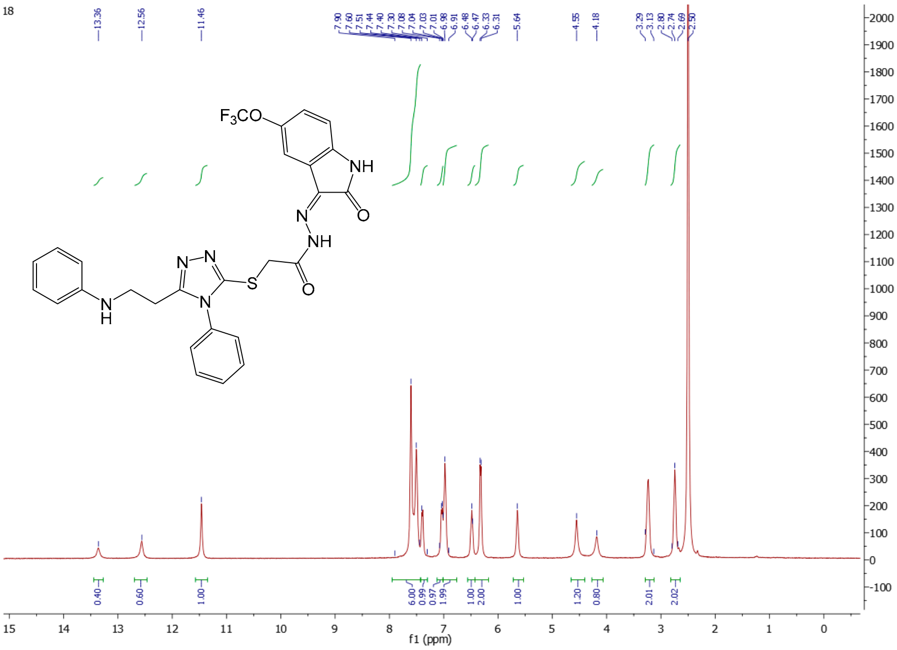

Figure S22. ^{1}H NMR (400 MHz, DMSO-d6) spectrum of **8**

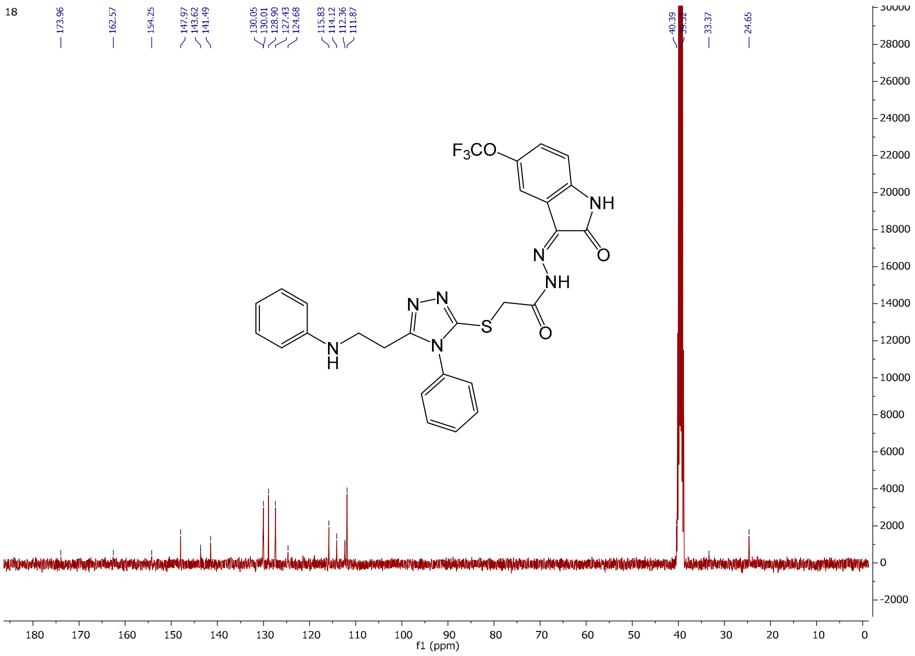

Figure S23. ^{13}C NMR (101 MHz, DMSO-d6) spectrum of **8**

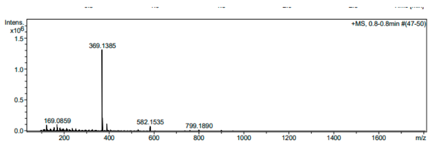

Meas. m/z	#	Formula	Score	m/z	err [ppm]	Mean err [ppm]	mSigma	rdb	e⁻ Conf	N-Rule
582.1535	1	C 23 H 17 F N 17 S	46.86	582.1552	3.0	2.7	9.2	23.5	even	ok
	2	C 28 H 25 F N 3 O 10	49.81	582.1518	-2.8	-1.9	9.9	17.5	even	ok
	3	C 25 H 20 N 13 O 3 S	80.43	582.1527	-1.3	-1.5	12.7	22.5	even	ok
	4	C 29 H 21 F N 7 O 6	100.00	582.1532	-0.5	0.4	13.0	22.5	even	ok
	5	C 27 H 23 F 3 N 7 O 3 S	88.56	582.1530	-0.9	-1.1	14.0	18.5	even	ok
	6	C 26 H 22 F 2 N 7 O 7	72.69	582.1543	1.5	2.4	15.0	18.5	even	ok
	7	C 26 H 27 F 3 N 3 O 7 S	37.12	582.1516	-3.2	-3.3	17.1	13.5	even	ok
	8	C 22 H 21 F N 13 O 4 S	87.43	582.1539	0.7	0.4	17.3	18.5	even	ok
	9	C 28 H 28 N 3 O 9 S	76.14	582.1541	1.0	0.9	19.2	16.5	even	ok
	10	C 26 H 13 F N 17	39.67	582.1518	-2.8	-2.0	20.9	28.5	even	ok
	11	[illegible]	[illegible]	[illegible]	[illegible]	[illegible]	[illegible]	[illegible]	[illegible]	[illegible]

Figure S24. HRMS spectrum of **8**

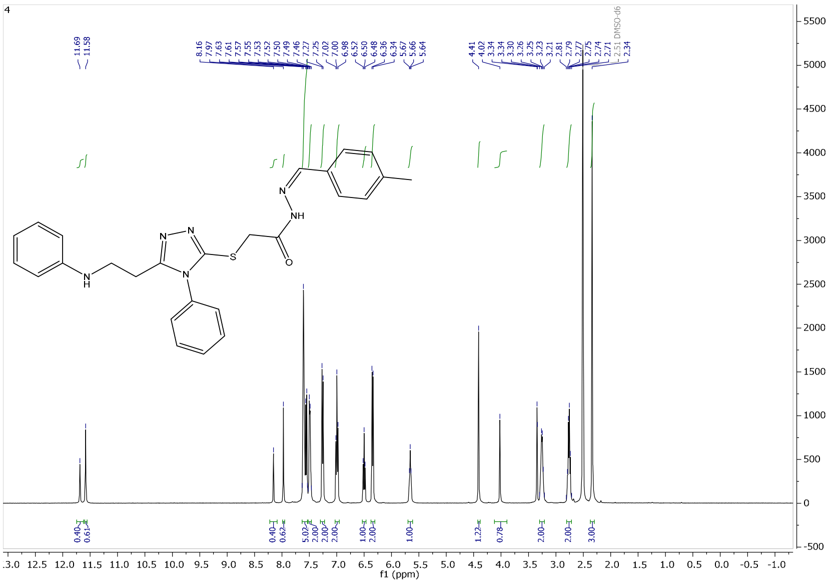

Figure S25. ^{1}H NMR (400 MHz, DMSO-d_6) spectrum of **9**

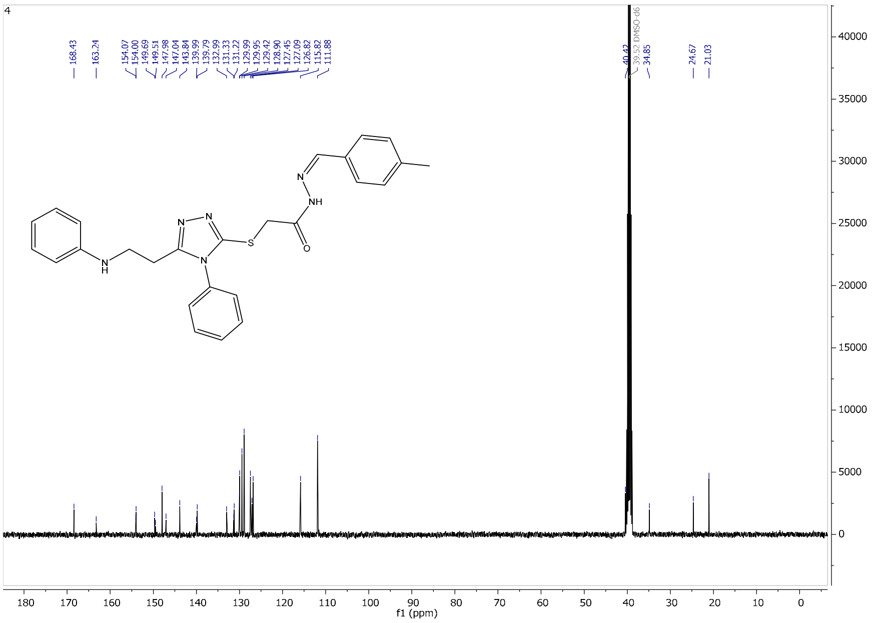

Figure S26. ^{13}C NMR (101 MHz, DMSO-d_6) spectrum of **9**

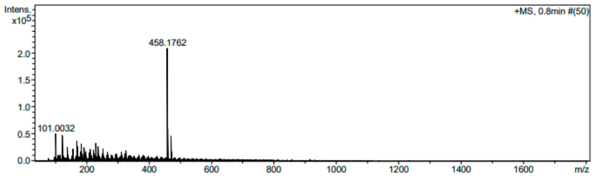

Meas. m/z	#	Formula	Score	m/z	err [ppm]	Mean err [ppm]	mSigma	rdb	e⁻ Conf	N-Rule
458.1762	1	C 24 H 24 N 7 O S	91.49	458.1758	-1.0	-1.4	7.6	16.5	even	ok
	2	C 26 H 29 N Na O 3 S	100.00	458.1760	-0.4	-0.7	9.6	12.5	even	ok
	3	C 23 H 28 N 3 O 5 S	35.64	458.1744	-3.9	-4.3	15.4	11.5	even	ok
	4	C 31 H 24 N O 3	44.31	458.1751	-2.5	-1.4	25.2	20.5	even	ok
471.1966	1	C 33 H 27 O 3	100.00	471.1955	-2.5	-1.5	34.6	20.5	even	ok
	2	C 26 H 27 N 6 O S	82.44	471.1962	-1.0	-0.1	54.2	16.5	even	ok
	3	C 28 H 32 Na O 3 S	85.55	471.1964	-0.4	0.6	57.0	12.5	even	ok

Figure S27. HRMS spectrum of **9**

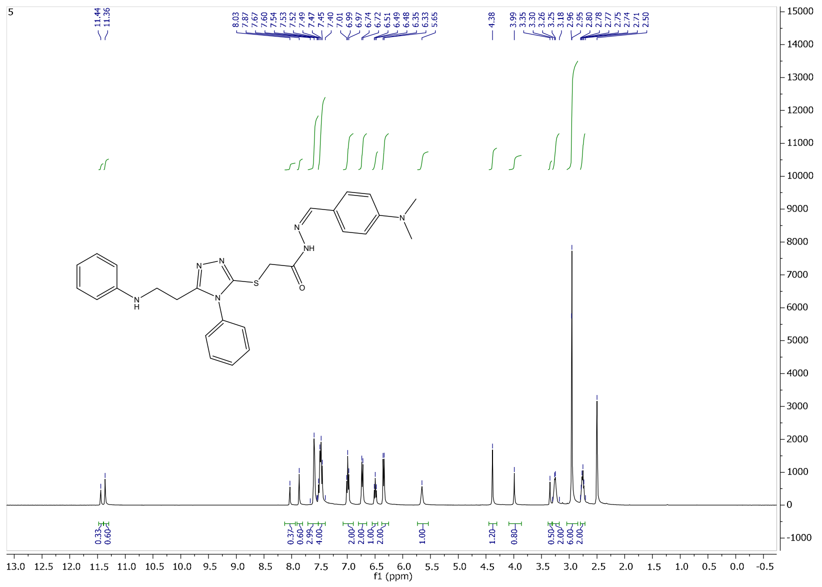

Figure S28. ^{1}H NMR (400 MHz, DMSO-d6) spectrum of **10**

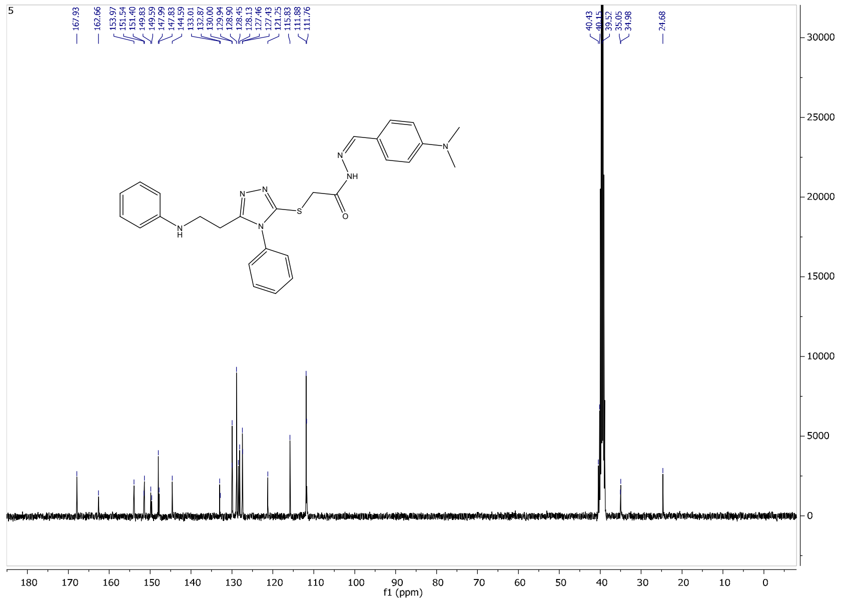

Figure S29. ^{13}C NMR (101 MHz, DMSO-d6) spectrum of **10**

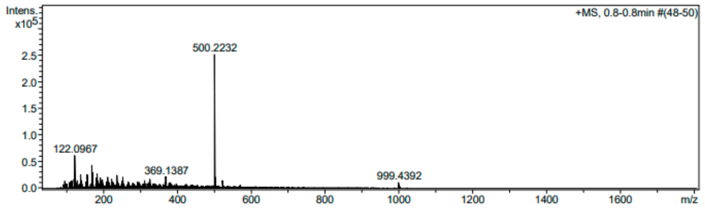

Meas. m/z	#	Formula	Score	m/z	err [ppm]	Mean err [ppm]	mSigma	rdb	e⁻ Conf	N-Rule
500.2232	1	C 27 H 30 N 7 O S	94.26	500.2227	-1.0	-1.4	6.1	16.5	even	ok
	2	C 29 H 35 N Na O 3 S	100.00	500.2230	-0.4	0.5	9.9	12.5	even	ok
	3	C 26 H 34 N 3 O 5 S	36.70	500.2214	-3.6	-4.0	13.6	11.5	even	ok
	4	C 34 H 30 N O 3	45.34	500.2220	-2.3	-1.1	24.1	20.5	even	ok

Figure S30. HRMS spectrum of **10**

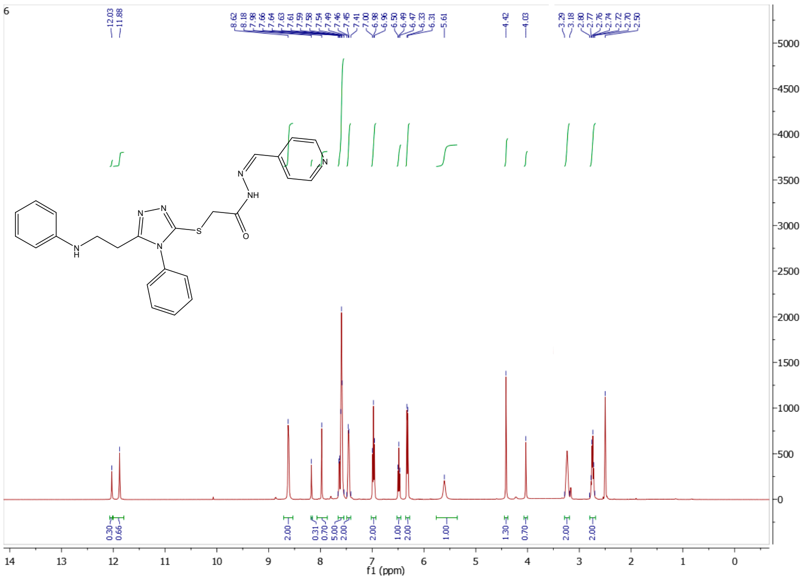

Figure S31. ^{1}H NMR (400 MHz, DMSO-d$_6$) spectrum of **11**

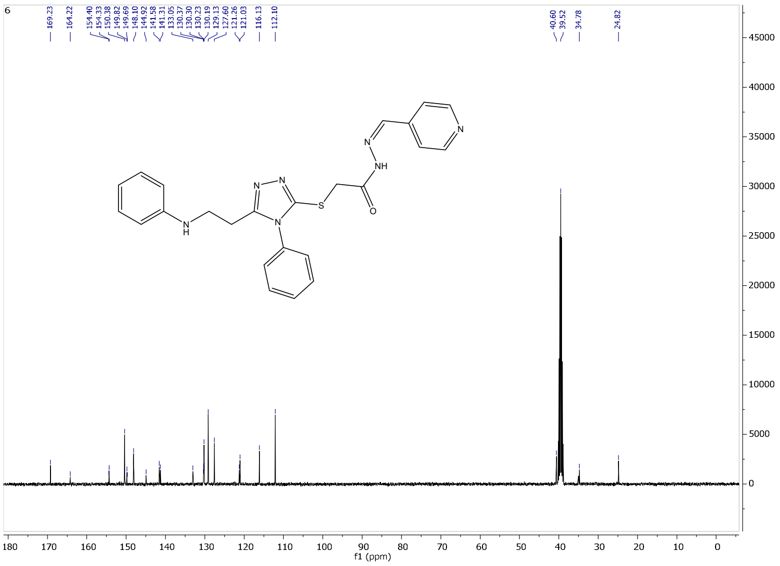

Figure S32. ^{13}C NMR (400 MHz, DMSO-d$_6$) spectrum of **11**

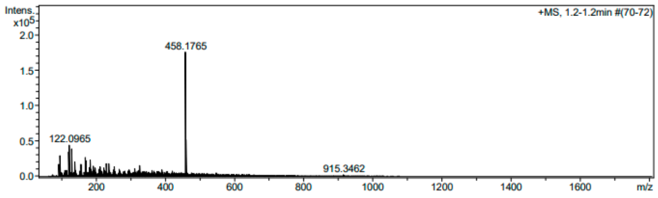

Meas. m/z	#	Formula	Score	m/z	err [ppm]	Mean err [ppm]	mSigma	rdb	e⁻ Conf	N-Rule
458.1765	1	C 24 H 24 N 7 O S	91.43	458.1758	-1.6	-1.7	9.8	16.5	even	ok
	2	C 26 H 29 N Na O 3 S	100.00	458.1760	-1.0	-1.1	12.4	12.5	even	ok
	3	C 28 H 28 N O 3 S	35.35	458.1784	4.3	4.2	18.0	15.5	even	ok
	4	C 31 H 24 N O 3	48.71	458.1751	-3.1	-1.9	21.3	20.5	even	ok
	5	C 20 H 24 N 7 O 6	25.00	458.1783	3.9	5.0	38.2	12.5	even	ok

Figure S33. HRMS spectrum of **11**

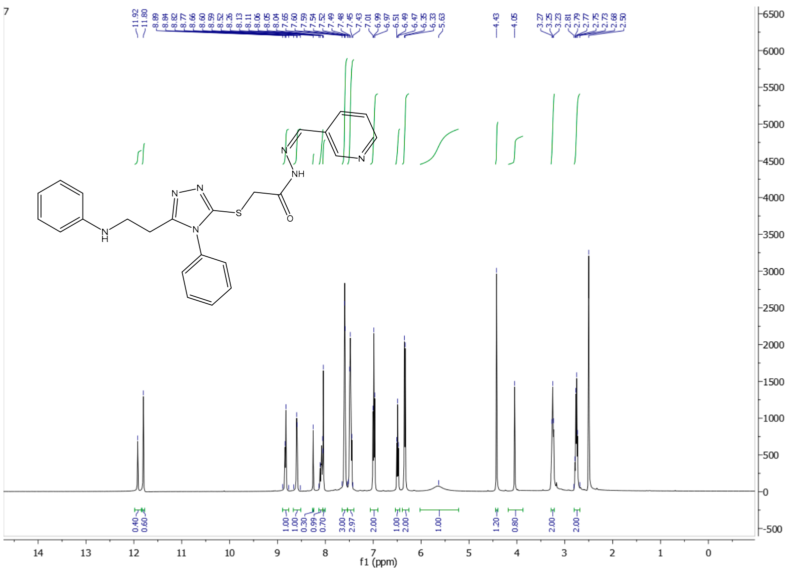

Figure S34. ^{1}H NMR (400 MHz, DMSO-d$_6$) spectrum of **12**

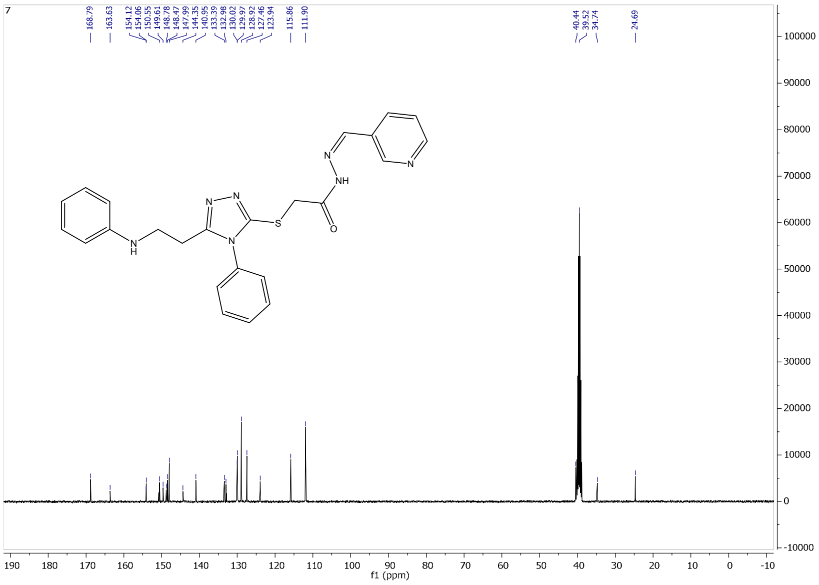

Figure S35. ^{13}C NMR (101 MHz, DMSO-d$_6$) spectrum of **12**

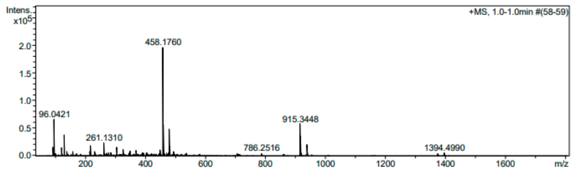

Meas. m/z	#	Formula	Score	m/z	err [ppm]	Mean err [ppm]	mSigma	rdb	e⁻ Conf	N-Rule
458.1760	1	C 24 H 24 N 7 O S	98.75	458.1758	-0.5	-0.6	9.2	16.5	even	ok
	2	C 26 H 29 N Na O 3 S	100.00	458.1760	0.1	0.0	11.9	12.5	even	ok
	3	C 23 H 28 N 3 O 5 S	43.88	458.1744	-3.4	-3.5	13.1	11.5	even	ok
	4	C 31 H 24 N O 3	47.92	458.1751	-2.0	-0.6	27.6	20.5	even	ok
	5	C 19 H 21 N 11 Na O 2	36.28	458.1772	2.7	3.7	31.4	14.5	even	ok
	6	C 19 H 28 N 3 O 10	38.24	458.1769	2.1	3.1	35.6	7.5	even	ok
480.1577	1	C 31 H 23 N Na O 3	100.00	480.1570	-1.5	-0.6	5.0	20.5	even	ok
	2	C 29 H 18 N 7 O	83.57	480.1567	-2.1	-1.3	6.6	24.5	even	ok
	3	C 33 H 22 N O 3	49.75	480.1594	3.5	4.4	10.5	23.5	even	ok
	4	C 24 H 23 N 7 Na O S	87.72	480.1577	-0.1	-0.3	27.0	16.5	even	ok
	5	C 25 H 26 N 3 O 5 S	48.49	480.1588	2.2	1.9	31.0	14.5	even	ok

Figure S36. HRMS spectrum of **12**

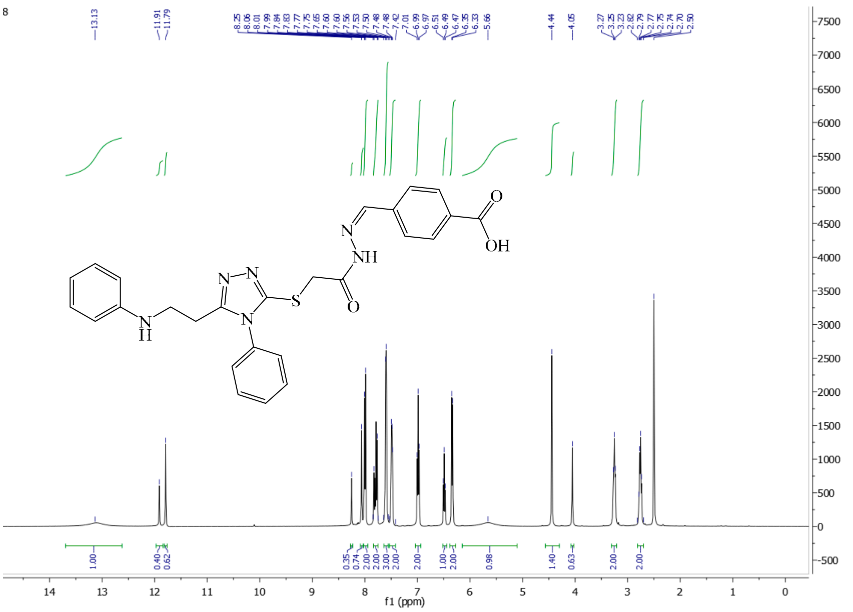

Figure S37. ^{1}H NMR (400 MHz, DMSO-d_6) spectrum of **13**

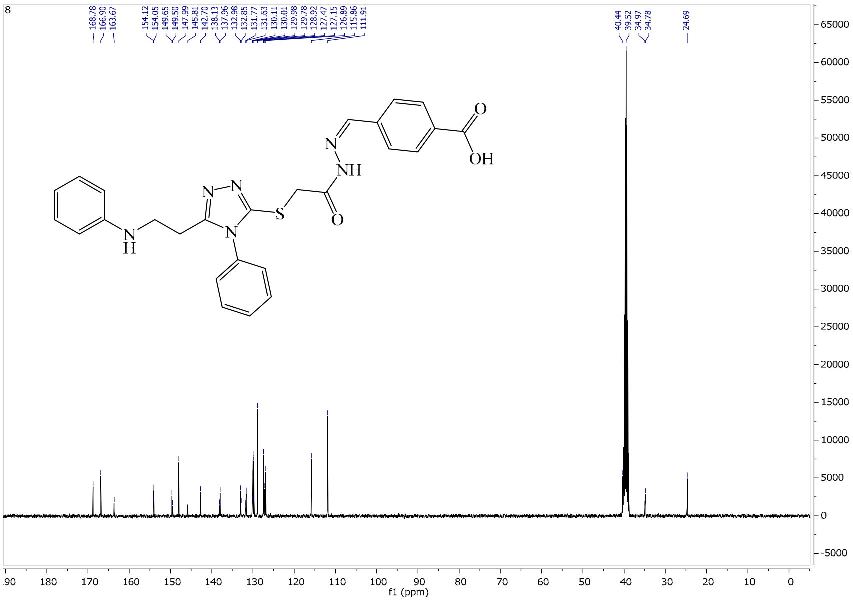

Figure S38. ^{13}C NMR (101 MHz, DMSO-d_6) spectrum of **13**

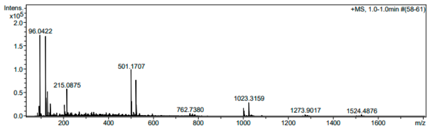

Meas. m/z	#	Formula	Score	m/z	err [ppm]	Mean err [ppm]	mSigma	rdb	e⁻ Conf	N-Rule
501.1707	1	C 29 H 26 N 4 Na O S	58.12	501.1720	2.5	3.4	11.6	18.5	even	ok
	2	C 26 H 25 N 6 O 3 S	92.74	501.1703	-0.7	-1.2	12.9	17.5	even	ok
	3	C 28 H 30 Na O 5 S	100.00	501.1706	-0.1	-0.6	15.2	13.5	even	ok
	4	C 33 H 25 O 5	59.25	501.1697	-2.1	-1.1	17.6	21.5	even	ok
	5	C 25 H 29 N 2 O 7 S	34.10	501.1690	-3.4	-3.8	23.9	12.5	even	ok
	6	C 34 H 21 N 4 O	67.43	501.1710	0.6	1.5	28.6	26.5	even	ok

Figure S39. HRMS spectrum of **13**

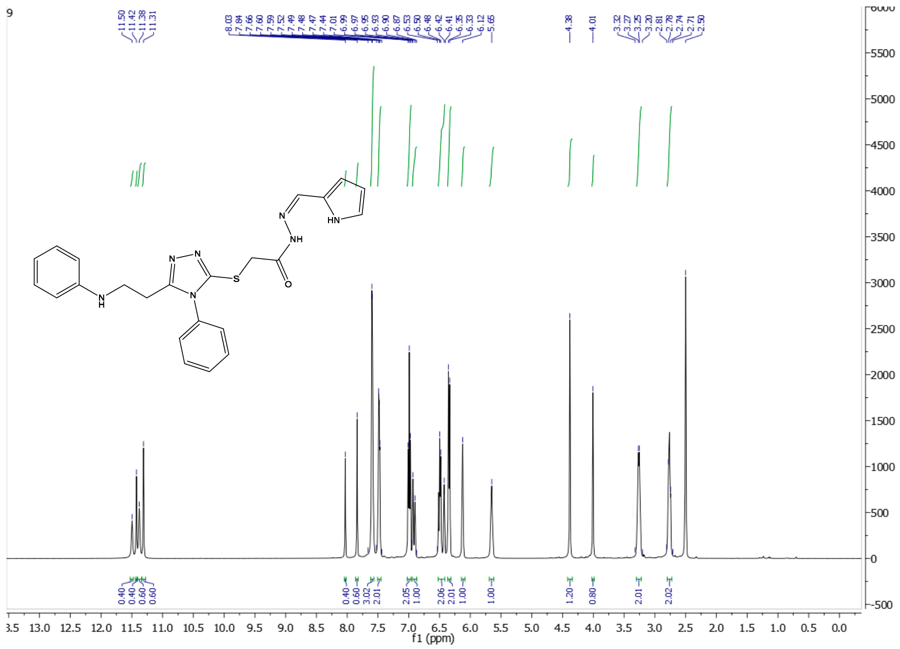

Figure S40. ^{1}H NMR (400 MHz, DMSO-d6) spectrum of **14**

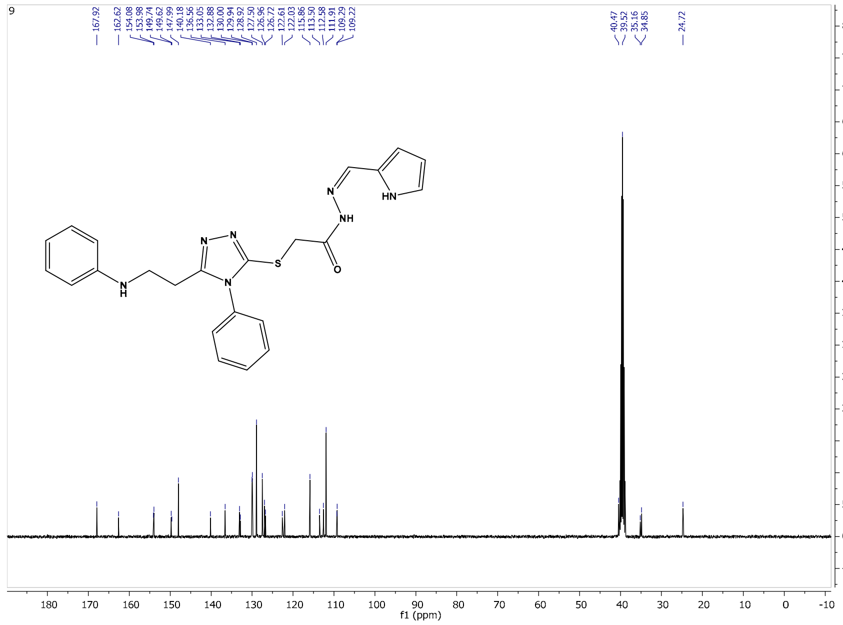

Figure S41. ^{13}C NMR (101 MHz, DMSO-d6) spectrum of **14**

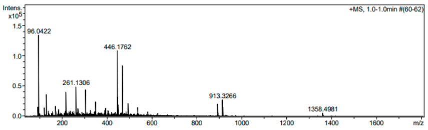

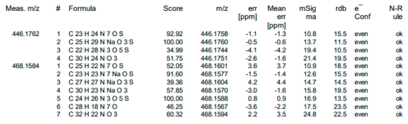

Meas. m/z	#	Formula	Score	m/z	err [ppm]	Mean err [ppm]	mSigma	rdb	e⁻ Conf	N-Rule
446.1762	1	C 23 H 24 N 7 O S	92.92	446.1758	-1.1	-1.3	10.8	15.5	even	ok
	2	C 25 H 29 N Na O 3 S	100.00	446.1760	-0.5	-0.6	13.7	11.5	even	ok
	3	C 22 H 28 N 3 O 5 S	34.99	446.1744	-4.1	-4.2	19.4	10.5	even	ok
	4	C 30 H 24 N O 3	51.75	446.1751	-2.6	-1.6	21.4	19.5	even	ok
468.1584	1	C 25 H 22 N 7 O S	52.05	468.1601	3.6	3.7	10.9	18.5	even	ok
	2	C 23 H 23 N 7 Na O S	91.60	468.1577	-1.5	-1.4	12.6	15.5	even	ok
	3	C 27 H 27 N Na O 3 S	39.36	468.1604	4.2	4.4	14.7	14.5	even	ok
	4	C 30 H 23 N Na O 3	57.85	468.1570	-3.0	-1.6	15.8	19.5	even	ok
	5	C 24 H 26 N 3 O 5 S	100.00	468.1588	0.8	0.9	16.9	13.5	even	ok
	6	C 28 H 18 N 7 O	46.25	468.1567	-3.6	-2.2	17.5	23.5	even	ok
	7	C 32 H 22 N O 3	60.32	468.1594	2.2	3.5	24.8	22.5	even	ok

Figure S42. HRMS spectrum of **14**

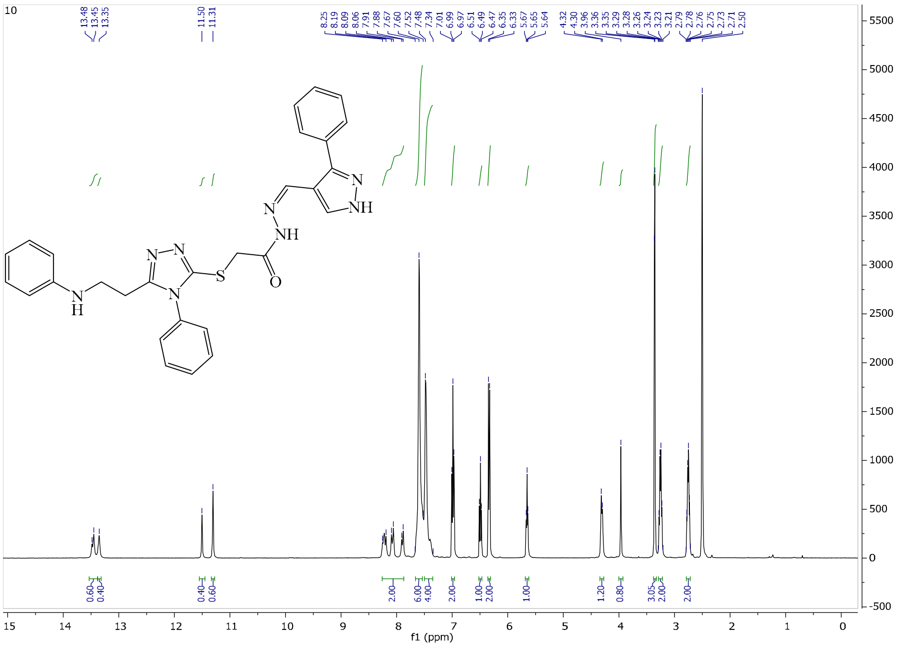

Figure S43. ^{1}H NMR (400 MHz, DMSO-d6) spectrum of **15**

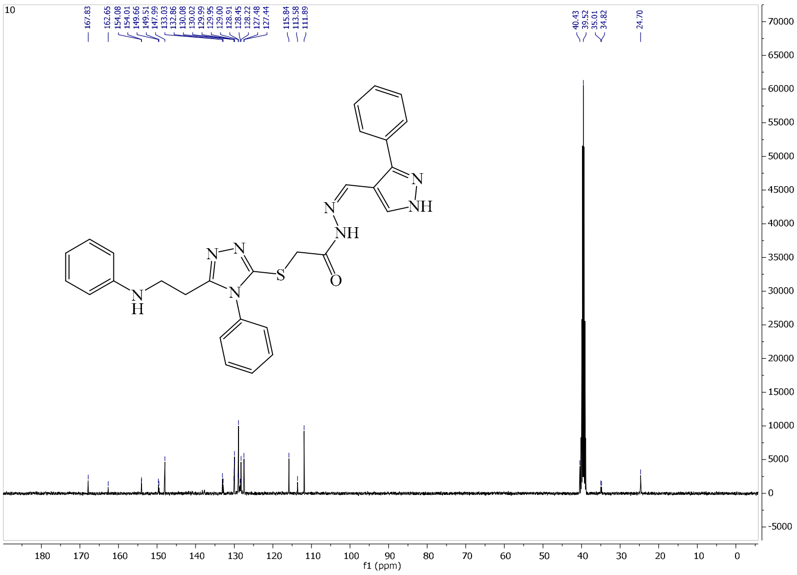

Figure S44. ^{13}C NMR (101 MHz, DMSO-d6) spectrum of **15**

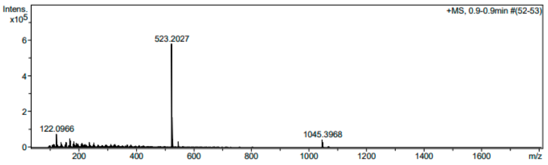

Meas. m/z	#	Formula	Score	m/z	err [ppm]	Mean err [ppm]	mSigma	rdb	e⁻ Conf	N-Rule
523.2027	1	C 23 H 31 N 4 O 10	91.35	523.2035	1.5	2.4	16.0	10.5	even	ok
	2	C 23 H 24 N 12 Na O 2	75.74	523.2037	2.0	2.7	17.9	17.5	even	ok
	3	C 27 H 31 N 4 O 5 S	47.12	523.2010	-3.3	-3.7	19.2	14.5	even	ok
	4	C 21 H 19 N 18	83.10	523.2035	1.4	2.2	20.8	21.5	even	ok
	5	C 22 H 28 N 8 Na O 6	100.00	523.2024	-0.6	0.2	22.6	12.5	even	ok
	6	C 20 H 23 N 14 O 4	83.91	523.2021	-1.1	-0.4	24.5	16.5	even	ok
	7	C 22 H 35 O 14	83.08	523.2021	-1.1	-0.4	25.2	5.5	even	ok
	8	C 21 H 32 N 4 Na O 10	43.35	523.2011	-3.1	-2.2	26.0	7.5	even	ok
	9	C 28 H 27 N 8 O S	86.95	523.2023	-0.8	-1.1	26.8	19.5	even	ok
	10	C 30 H 32 N 2 Na O 3 S	90.76	523.2026	-0.2	0.5	30.1	15.5	even	ok
	11	C 35 H 27 N 2 O 3	36.21	523.2016	-2.1	-1.0	46.4	23.5	even	ok
	12	C 38 H 28 Na O	32.68	523.2032	1.0	2.1	59.0	24.5	even	ok

Figure S45. HRMS spectrum of **15**

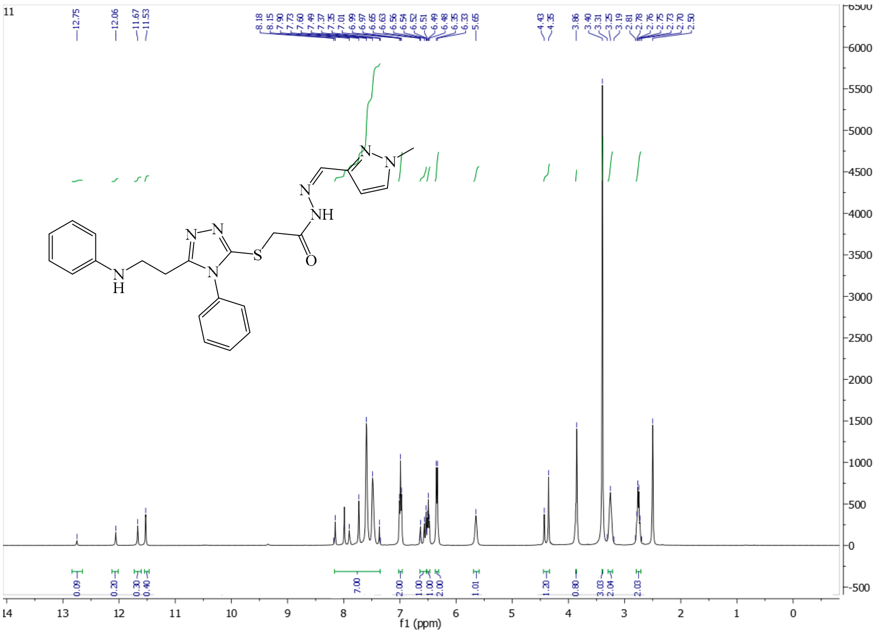

Figure S46. ^{1}H NMR (400 MHz, DMSO-d_6) spectrum of **16**

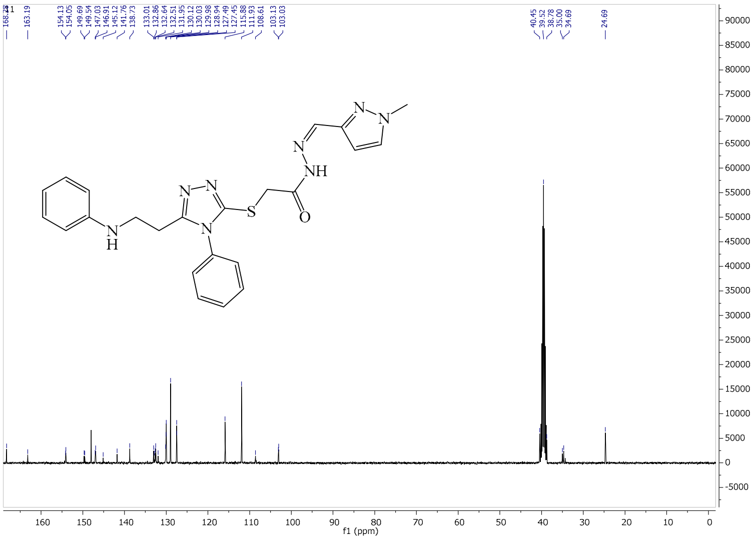

Figure S47. ^{13}C NMR (101 MHz, DMSO-d_6) spectrum of **16**

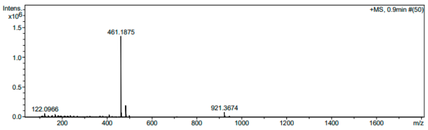

Meas. m/z	#	Formula	Score	m/z	err [ppm]	Mean err [ppm]	mSigma	rdb	e⁻ Conf	N-Rule
461.1875	1	C 21 H 30 N 2 Na O 8	43.57	461.1894	4.3	5.4	9.6	7.5	even	ok
	2	C 19 H 25 N 8 O 6	51.54	461.1892	3.7	4.7	11.5	11.5	even	ok
	3	C 18 H 22 N 12 Na O 2	92.41	461.1881	1.4	2.3	14.5	13.5	even	ok
	4	C 16 H 17 N 18	100.00	461.1878	0.8	1.7	17.2	17.5	even	ok
	5	C 18 H 29 N 4 O 10	97.31	461.1878	0.8	1.7	18.3	6.5	even	ok
	6	C 17 H 26 N 8 Na O 6	74.54	461.1868	-1.5	-0.6	22.7	8.5	even	ok
	7	C 23 H 25 N 8 O S	67.62	461.1867	-1.8	-1.8	24.8	15.5	even	ok
	8	C 25 H 30 N 2 Na O 3 S	74.78	461.1869	-1.1	-1.1	26.6	11.5	even	ok
	9	C 17 H 33 O 14	57.81	461.1865	-2.1	-1.2	27.4	1.5	even	ok
	10	C 27 H 29 N 2 O 3 S	25.25	461.1893	4.1	4.1	37.2	14.5	even	ok
	11	C 30 H 25 N 2 O 3	28.45	461.1860	-3.2	-2.0	42.7	19.5	even	ok
	12	C 33 H 26 Na O	40.61	461.1876	0.3	1.5	55.3	20.5	even	ok
483.1691	1	C 23 H 24 N 8 Na O S	100.00	483.1686	-1.1	-1.3	7.5	15.5	even	ok
	2	C 24 H 27 N 4 O 5 S	92.95	483.1697	1.1	0.9	11.3	13.5	even	ok
	3	C 25 H 23 N 8 O S	38.42	483.1710	3.9	3.6	14.7	18.5	even	ok
	4	C 22 H 28 N 4 Na O 5 S	38.22	483.1673	-3.9	-4.1	15.3	10.5	even	ok
	5	C 23 H 31 O 9 S	71.75	483.1683	-1.7	-1.8	17.6	8.5	even	ok
	6	C 30 H 24 N 2 Na O 3	47.89	483.1679	-2.5	-1.4	25.2	19.5	even	ok
	7	C 28 H 19 N 8 O	38.75	483.1676	-3.1	-2.1	26.7	23.5	even	ok
	8	C 32 H 23 N 2 O 3	38.66	483.1703	2.5	3.5	34.7	22.5	even	ok
	9	C 19 H 24 N 8 Na O 6	21.22	483.1711	4.1	5.1	37.3	11.5	even	ok

Figure S48. HRMS spectrum of **16**

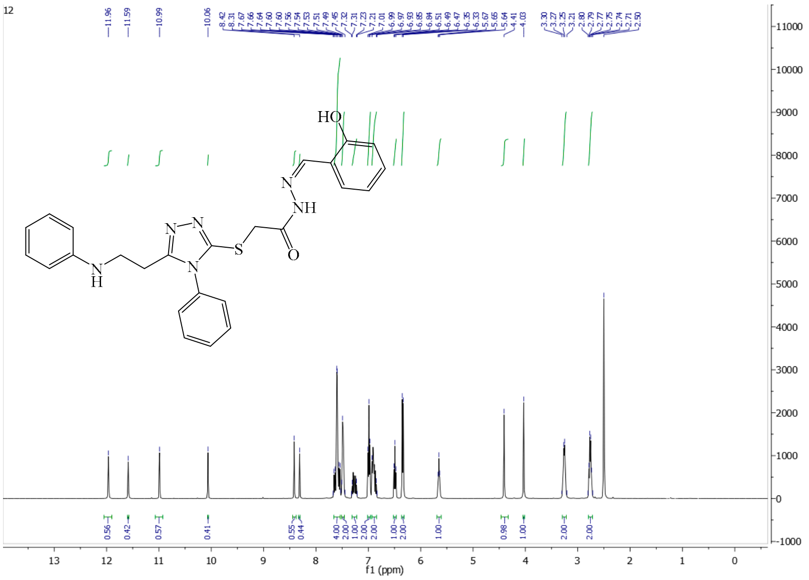

Figure S49. ^{1}H NMR (400 MHz, DMSO-d$_6$) spectrum of **17**

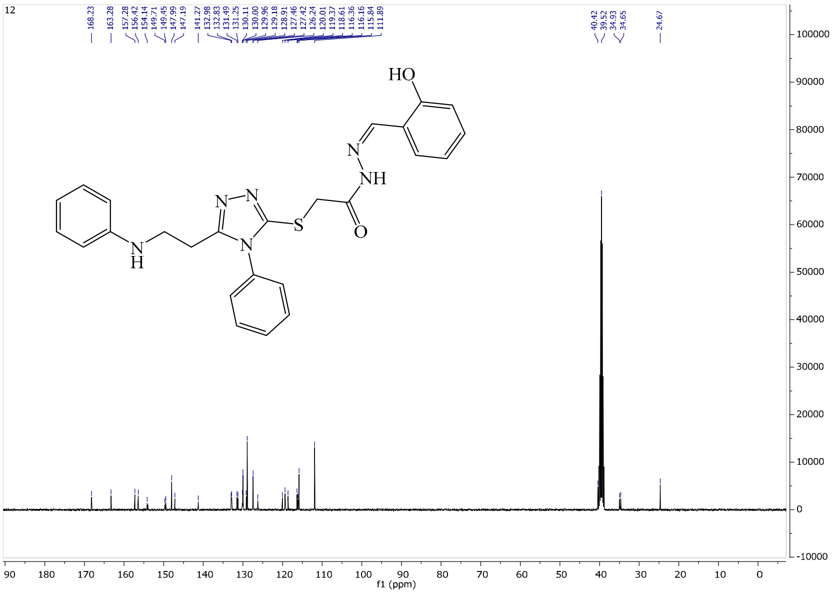

Figure S50. ^{13}C NMR (101 MHz, DMSO-d$_6$) spectrum of **17**

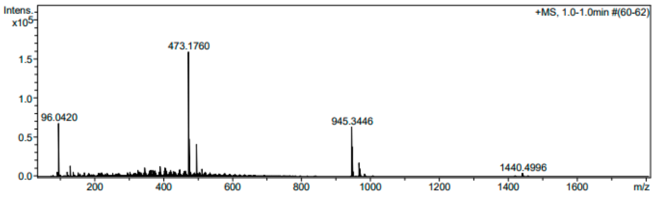

Meas. m/z	#	Formula	Score	m/z	err [ppm]	Mean err [ppm]	mSigma	rdb	e⁻ Conf	N-Rule
473.1760	1	C 25 H 25 N 6 O 2 S	91.08	473.1754	-1.1	-1.3	10.9	16.5	even	ok
	2	C 27 H 30 Na O 4 S	100.00	473.1757	-0.5	-0.7	12.8	12.5	even	ok
	3	C 28 H 26 N 4 Na S	60.95	473.1770	2.3	2.1	16.4	17.5	even	ok
	4	C 24 H 29 N 2 O 6 S	34.65	473.1741	-4.0	-4.1	18.5	11.5	even	ok
	5	C 32 H 25 O 4	50.32	473.1747	-2.6	-1.2	21.6	20.5	even	ok
	6	C 33 H 21 N 4	65.63	473.1761	0.3	1.5	34.2	25.5	even	ok
	7	C 21 H 25 N 6 O 7	21.62	473.1779	4.2	5.2	35.6	12.5	even	ok
495.1574	1	C 32 H 24 Na O 4	73.41	495.1567	-1.4	-0.3	16.5	20.5	even	ok
	2	C 23 H 19 N 12 S	89.01	495.1571	-0.6	-1.0	16.7	20.5	even	ok
	3	C 25 H 24 N 6 Na O 2 S	100.00	495.1574	-0.1	-0.4	16.9	16.5	even	ok
	4	C 30 H 19 N 6 O 2	60.88	495.1564	-2.0	-0.9	18.2	24.5	even	ok
	5	C 26 H 27 N 2 O 6 S	58.17	495.1584	2.1	1.8	19.2	14.5	even	ok
	6	C 34 H 23 O 4	36.29	495.1591	3.4	4.6	21.8	23.5	even	ok
	7	C 33 H 20 N 4 Na	64.28	495.1580	1.3	2.4	24.9	25.5	even	ok
	8	C 24 H 28 N 2 Na O 6 S	38.31	495.1560	-2.8	-3.0	28.9	11.5	even	ok

Figure S51. HRMS spectrum of **17**

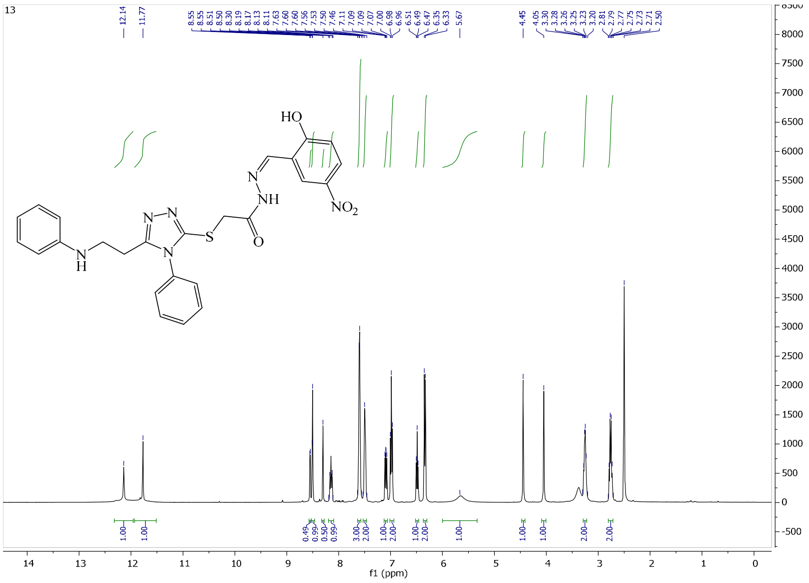

Figure S52. ^{1}H NMR (400 MHz, DMSO-d$_6$) spectrum of **18**

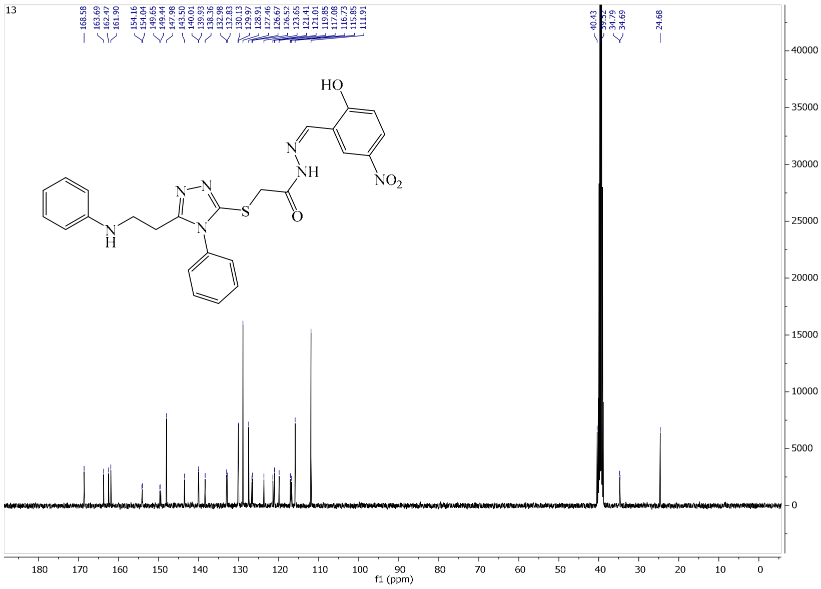

Figure S53. ^{13}C NMR (101 MHz, DMSO-d$_6$) spectrum of **18**

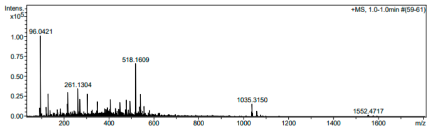

Meas. m/z	#	Formula	Score	m/z	err [ppm]	Mean err [ppm]	mSigma	rdb	e⁻ Conf	N-Rule
518.1609	1	C 24 H 21 N 11 Na S	50.62	518.1594	-2.9	-3.2	8.9	19.5	even	ok
	2	C 25 H 24 N 7 O 4 S	91.77	518.1605	-0.8	-1.1	9.5	17.5	even	ok
	3	C 27 H 29 N Na O 6 S	100.00	518.1608	-0.3	-0.6	11.6	13.5	even	ok
	4	C 26 H 20 N 11 S	69.29	518.1618	1.7	1.4	12.0	22.5	even	ok
	5	C 28 H 25 N 5 Na O 2 S	57.12	518.1621	2.3	2.0	13.6	18.5	even	ok
	6	C 24 H 28 N 3 O 8 S	34.46	518.1592	-3.4	-3.7	19.0	12.5	even	ok
	7	C 32 H 24 N O 6	47.67	518.1598	-2.2	-1.0	23.7	21.5	even	ok
	8	C 33 H 20 N 5 O 2	58.80	518.1612	0.4	1.6	34.5	26.5	even	ok
	9	C 36 H 21 N 3 Na	17.12	518.1628	3.5	4.7	45.5	27.5	even	ok
540.1419	1	C 24 H 27 N 3 Na O 8 S	80.89	540.1411	-1.5	-1.7	10.0	12.5	even	ok
	2	C 22 H 22 N 9 O 6 S	68.71	540.1408	-2.0	-2.3	10.4	16.5	even	ok
	3	C 26 H 26 N 3 O 8 S	48.43	540.1435	3.0	2.7	11.4	15.5	even	ok
	4	C 25 H 23 N 7 Na O 4 S	88.93	540.1424	1.0	0.7	12.3	17.5	even	ok
	5	C 23 H 18 N 13 O 2 S	100.00	540.1422	0.5	0.2	12.9	21.5	even	ok
	6	C 26 H 19 N 11 Na S	31.58	540.1438	3.5	3.2	22.9	22.5	even	ok
	7	C 29 H 22 N 3 O 8	31.47	540.1401	-3.3	-2.0	26.4	20.5	even	ok
	8	C 32 H 23 N Na O 6	63.01	540.1418	-0.3	1.0	35.3	21.5	even	ok
	9	C 30 H 18 N 7 O 4	54.04	540.1415	-0.8	0.4	36.4	25.5	even	ok
	10	C 20 H 27 N 3 Na O 13	24.96	540.1436	3.1	4.0	37.6	8.5	even	ok
	11	C 29 H 15 N 11 Na	26.97	540.1404	-2.8	-1.6	39.8	27.5	even	ok
	12	C 33 H 19 N 5 Na O 2	27.00	540.1431	2.2	3.4	46.8	26.5	even	ok

Figure S54. HRMS spectrum of **18**

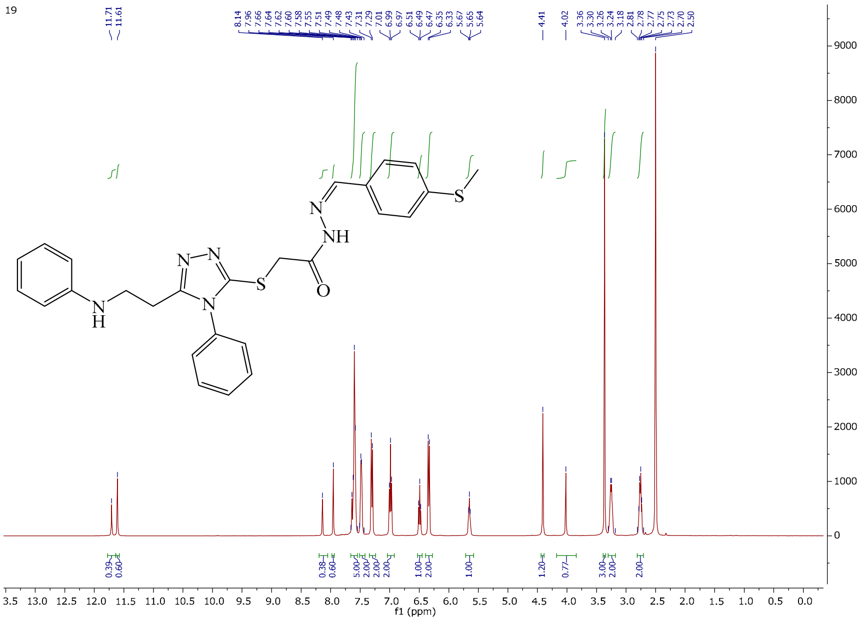

Figure S55. ^{1}H NMR (400 MHz, DMSO-d_6) spectrum of **19**

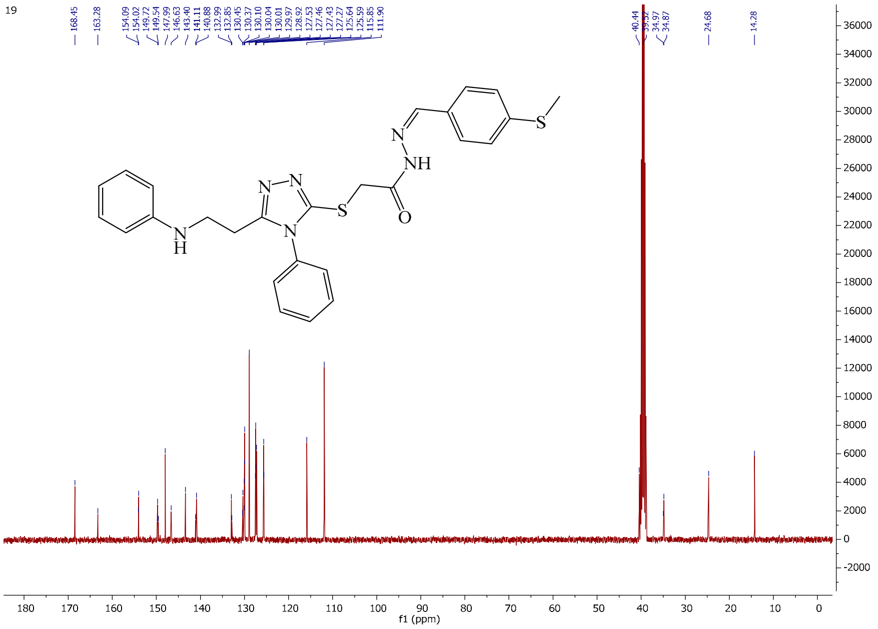

Figure S56. ^{13}C NMR (101 MHz, DMSO-d_6) spectrum of **19**

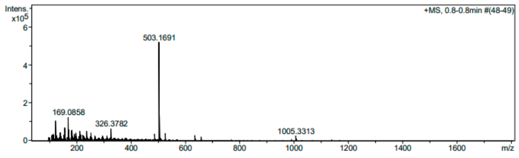

Meas. m/z	#	Formula	Score	m/z	err [ppm]	Mean err [ppm]	mSigma	rdb	e⁻ Conf	N-Rule
503.1691	1	C 21 H 31 N 2 O 10 S	100.00	503.1694	0.6	0.6	5.4	7.5	even	ok
	2	C 22 H 27 N 6 O 6 S	44.38	503.1707	3.2	3.2	8.7	12.5	even	ok
	3	C 18 H 23 N 12 O 4 S	61.47	503.1680	-2.1	-2.2	10.3	13.5	even	ok
	4	C 19 H 19 N 16 S	85.97	503.1694	0.5	0.5	13.8	18.5	even	ok
	5	C 25 H 23 N 6 O 6	24.01	503.1674	-3.5	-2.0	33.0	17.5	even	ok
	6	C 29 H 27 O 8	38.60	503.1700	1.9	3.7	34.6	16.5	even	ok
	7	C 26 H 27 N 6 O S 2	35.55	503.1682	-1.7	-1.8	38.9	16.5	even	ok
	8	C 26 H 19 N 10 O 2	38.61	503.1687	-0.8	0.6	44.5	22.5	even	ok
	9	C 30 H 31 O 3 S 2	15.32	503.1709	3.6	3.6	47.9	15.5	even	ok
	10	C 33 H 27 O 3 S	14.08	503.1675	-3.1	-1.7	56.0	20.5	even	ok

Figure S57. HRMS spectrum of **19**

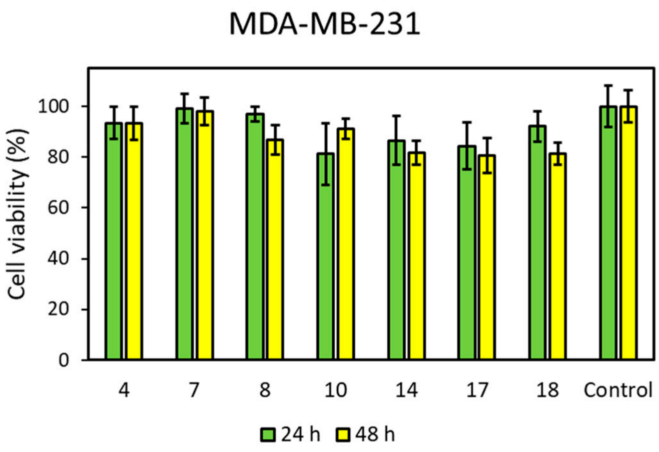

Figure S58. The effect of 10 μM compound concentration on MDA-MB-231 cell line viability after 24 and 48 h.

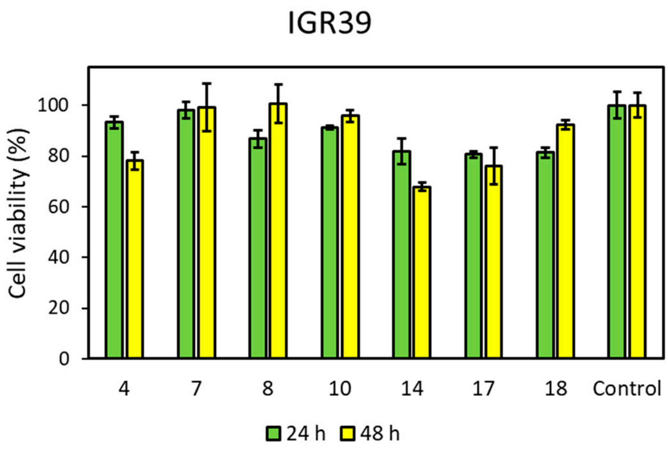

Figure S59. The effect of 10 μM compound concentration on IGR39 cell line viability after 24 and 48 h.

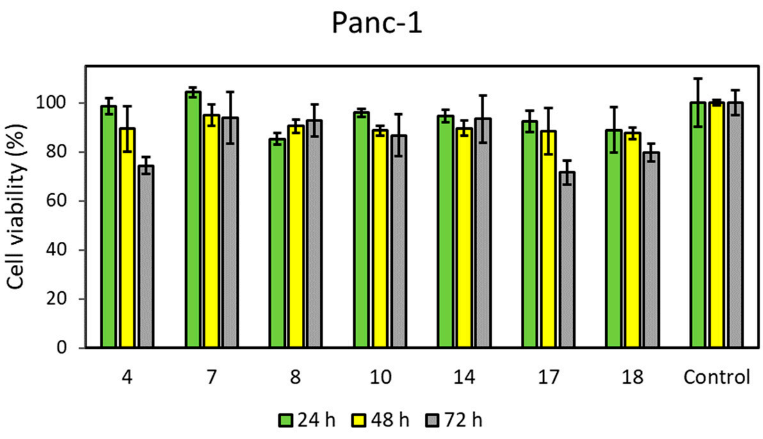

Figure S60. The effect of 10 μM compound concentration on Panc-1 cell line viability after 24, 48, and 72 h.

Procedure S1. Evaluation of 10 μM compound concentration effect on cell line viability.

The effect of 10 μM compounds on cell viability was studied using 3-(4,5-dimethylthiazol-2-yl)-2,5-diphenyltetrazolium bromide (MTT; Sigma-Aldrich Co., St Louis, MO, USA) assay. IGR39, MDA-MB-231, and Panc-1 cells were seeded in 96-well plates (Corning) in triplicates at a volume of 100 μL at 4 × 10^3 cells/well). After 24 h, the cells were treated with 10 μM of compounds **4**, **7**, **8**, **10**, **14**, **17**, and **18**. After 24 h, 48 h and 72 h (only Panc-1 cells), the MTT reagent has been added and cells were incubated for 4 h. Then the medium was aspirated, and the formed formazan crystals were dissolved in 100 μL DMSO (Sigma-Aldrich Co., St. Louis, MO, USA). The absorbance was measured at 570 and 630 nm using a multi-detection microplate reader. Compound effect on cell viability was calculated according to a formula:

$$Relative\ cell\ viability\ (\%) = \frac{A - A_0}{A_{NC} - A_0}$$

Where:
A – mean of absorbance of tested compound,
A_0 – mean of absorbance of blank (no cells, positive control),
A_{NC} – mean of absorbance of negative control (only cells, no treatment).
